# Identification of *Bacillus anthracis*, *Brucella* spp., and *Coxiella burnetii* DNA signatures from bushmeat

**DOI:** 10.1038/s41598-021-94112-9

**Published:** 2021-07-21

**Authors:** Robab Katani, Megan A. Schilling, Beatus Lyimo, Ernest Eblate, Andimile Martin, Triza Tonui, Isabella M. Cattadori, Stephen C. Francesconi, Anna B. Estes, Dennis Rentsch, Sreenidhi Srinivasan, Samson Lyimo, Lidia Munuo, Christian K. Tiambo, Francesca Stomeo, Paul Gwakisa, Fausta Mosha, Peter J. Hudson, Joram J. Buza, Vivek Kapur

**Affiliations:** 1 Applied Biological and Biosecurity Research Laboratory, Pennsylvania State University, University Park, PA USA; 2 The Huck Institutes of the Life Sciences, Pennsylvania State University, University Park, PA USA; 3 Department of Animal Science, Pennsylvania State University, University Park, PA USA; 4 Nelson Mandela African Institution of Science and Technology, Arusha, Tanzania; 5 Tanzania Wildlife Research Institute, Arusha, Tanzania; 6 Biosciences Eastern and Central Africa-International Livestock Research Institute (BecA-ILRI) Hub, Nairobi, Kenya; 7 Department of Biology, Pennsylvania State University, University Park, PA USA; 8 Naval Medical Research Center, Fort Detrick, MD USA; 9 Lincoln Park Zoo, Chicago, IL USA; 10 Sokoine University of Agriculture, Morogoro, Tanzania; 11 Ministry of Health Community Development Gender Elderly and Children, Dar es Salaam, Tanzania; 12 Present Address: Department of Environmental Studies, Carleton College, Northfield, MN USA; 13 Present Address: The European Molecular Biology Laboratory (EMBL), Heidelberg, Germany

**Keywords:** Infectious-disease diagnostics, Pathogens

## Abstract

Meat from wildlife species (bushmeat) represents a major source of dietary protein in low- and middle-income countries where humans and wildlife live in close proximity. Despite the occurrence of zoonotic pathogens in wildlife, their prevalence in bushmeat remains unknown. To assess the risk of exposure to major pathogens in bushmeat, a total of 3784 samples, both fresh and processed, were collected from three major regions in Tanzania during both rainy and dry seasons, and were screened by real-time PCR for the presence of DNA signatures of *Bacillus anthracis (B. anthracis)*, *Brucella* spp. (*Brucella*) and *Coxiella burnetii* (*Coxiella*). The analysis identified DNA signatures of *B. anthracis* (0.48%), *Brucella* (0.9%), and *Coxiella* (0.66%) in a total of 77 samples. Highest prevalence rates of *B. anthracis*, *Brucella*, and *Coxiella* were observed in wildebeest (56%), dik-dik (50%), and impala (24%), respectively. Fresh samples, those collected during the rainy season, and samples from Selous or Serengeti had a greater relative risk of being positive. Microbiome characterization identified *Firmicutes* and *Proteobacteria* as the most abundant phyla. The results highlight and define potential risks of exposure to endemic wildlife diseases from bushmeat and the need for future investigations to address the public health and emerging infectious disease risks associated with bushmeat harvesting, trade, and consumption.

## Introduction

“Bushmeat”, the meat and organs derived from harvested wildlife species, is a common source of animal protein throughout the tropics of the Americas, Asia, Australia, and Africa. In Tanzania, although bushmeat hunting is unlawful without permits, illegal wildlife poaching is a common practice year-round^1^. Bushmeat is not only a source of food and income for the poachers, their families, and communities, but is also used in many cultural, social, and religious practices^2^. A recent survey in the regions surrounding the Greater Serengeti ecosystem revealed that between 2 and 5 meals per week in a household contained bushmeat^3^. While the majority of bushmeat is consumed locally, a considerable amount is smuggled illegally into the United States and Western Europe. Reports suggest that up to 5 tons of bushmeat is smuggled via a single airport in Western Europe each week^4^. Bushmeat smuggling from Africa and elsewhere could act as a conduit for pathogen spread as suggested by the recent discovery of retroviruses and herpesviruses in bushmeat illegally imported to the US^5^. This is of particular relevance in light of the current global pandemic of SARS-CoV-2, and the increasing risk of newly emerging infectious diseases of zoonotic origin^6^ . It is widely recognized that 60% of emerging infectious diseases are zoonotic in origin, and of these 70% originate in wildlife^7^. In this study, we shall examine the prevalence of endemic *Bacillus anthracis* (*B. anthracis*), *Brucella* spp. (*Brucella*), and *Coxiella burnetii* (*Coxiella*) in bushmeat samples collected within Tanzania.

*Bacillus anthracis* is a spore-forming, Gram-positive bacterium and the causative agent of anthrax, a zoonotic disease that mostly affects grazing livestock and wild ungulates and poses a threat to human health. Studies have estimated that 20,000 to 100,000 human cases of anthrax occur worldwide annually^8,9^. The disease is endemic in poor rural areas, especially where there are extensive interactions between human, livestock and wildlife^8,9^. Outbreaks are regularly reported with an early event in 1962, causing mortality in 1200 impalas in Tanzania^10^. More recently, an outbreak in 2016–2017 in Northern Tanzania recorded positive cases in 131 wildlife and 39 livestock carcasses that were also used for consumption^8,10^. The recurrence of outbreaks in Tanzania are mainly the result of interactions between humans, wildlife, and livestock at specific “hotspots”, where both domestic and wild animals are potential sources of infections to humans^11^. People become infected when handling and consuming infected livestock and also bushmeat. Periods of drought facilitate anthrax outbreaks since they lead to aggregations of animals close to water sources, heavy overgrazing that exposes soil, and atypical migration patterns^12^.

*Brucella*, a genus of Gram-negative bacteria, is the causative agent of a number of zoonotic infections including brucellosis. Infection passes to humans through direct contact with infected animals, by the consumption of unpasteurized dairy products (often raw milk), or via occupational contact with infected animals as well as by the inhalation of airborne agents^13^. In sub-Saharan Africa, including Tanzania, studies have indicated the presence of *Brucella* in wildlife and that livestock to humans is considered an endemic problem causing devastating economic losses and challenges for public health^14–17^. A recent study in Kenya on *Brucella* suggested that transmission to humans increased as a consequence of close contact between livestock and wildlife^18^. Another study from Tanzania identified geographical “hotspots” with high anti-*Brucella* antibodies in humans, cattle, goats, and buffalos^14^. Other studies have emphasized that transmission of *Brucella* to humans was caused by the preparation and consumption of bushmeat^19–21^.

*Coxiella burnetii*, a Gram-negative bacterial CDC select agent that causes Q fever is also transmitted to livestock, wildlife, and humans via byproducts and the direct contact with infected animals. There are few studies on the prevalence of Q fever in Tanzania. One study on humans, livestock and wildlife found that the incidence of Q fever in cattle and wildlife was associated with arthropod vectors^22^. Another cross sectional sero-epidemiological study conducted in South Africa concluded higher risk of zoonotic transmission and economic loss was associated with the livestock-wildlife interface^23^ and this has been supported by a number of studies across Africa^21,24,25^.

Metagenomics has transformed microbiology by leading the way for a cultivation-independent determination of microbial profiles in samples, especially in samples similar to bushmeat that are subjected to a supply chain before consumption. Recent microbiome analyses have provided preliminary evidence for the presence of select agents in bushmeat samples^21^, and suggest that characterization of the microbiomes of bushmeat samples with evidence of DNA signatures for the three select agents may help better assess the overall microbial burden and potential public health risks associated with bushmeat harvest, handling, and consumption.

Thus, to better assess the prevalence of select agents and risks associated with the handling and consumption of bushmeat, we screened a total of 3784 fresh and processed bushmeat samples representing 42 animal species from the Serengeti, Ruaha, and Selous regions in Tanzania collected during rainy and dry seasons over a 2-year period for the presence of DNA signatures of *B. anthracis*, *Brucella* spp*.*, and *Coxiella*. Taken together, the results highlight the presence of select agent signatures and help define potential risks of exposure to endemic wildlife diseases from bushmeat in the country.

## Methods

### Study area

The sampling was carried out at three study savannah dominant sites in Tanzania: the Serengeti National Park (Serengeti), the Ruaha National Park (Ruaha), and the Selous Game Reserve (Selous). The ~ 25,000 km^2^ Serengeti ecosystem is located in northern Tanzania, bordering southern parts of Kenya^26^. It consists of several protected areas including the Serengeti National Park (14,760 km^2^, between 1° and 3° 30S and 34° and 36° E) and adjacent Ngorongoro Conservation Area, Game Reserves and Game Controlled Areas. Serengeti typically has a dry season (June-October) and a wet season (November–May). The Ruaha ecosystem (7° 30′ S 35° 00′ E), located in the southern region of Tanzania and at 20,226 km^2^, is comprised of Ruaha National Park and surrounding Game Reserves and Wildlife Management Areas. Ruaha typically has a dry season (May–October) and a wet season (November–April). The Selous Game Reserve (9° 0′ S 37° 24′ E) 50,000 km^2^ (partitioned to form Nyerere National Park, the largest Park in the country) is another protected area in Southern Tanzania. It is contiguous with the Mikumi National Park and Udzungwa Mountains National Park and contains one third of the abundant wildlife of Tanzania. Selous typically has a dry season (June–October) and a wet season (November–May). Typically, Tanzania has a short dry season (January–February) and a long dry season (June-October) and a short rainy season (November–December) and a long rainy season (March–May), although variations are observed in different regions^27–30^. For the purpose of this study, we considered the dry season from June-October while the rainy season was considered from the months of November–May for all the regions in the study.

### Bushmeat sample collection

Sample collection occurred over a two-year period between July 2016 and July 2018, from both dry and rainy seasons. Samples were identified as either “fresh” or “processed”. Fresh samples were those that appeared to be recently harvested while the processed samples included those that were either heavily salted, sun or air-dried, semi-boiled, or a combination of methods that resulted in dehydrated meat samples^31^. The samples were collected by teams of veterinarians, anti-poaching units, and local enumerator networks from villages surrounding the protected areas, where bushmeat is sold^32^. All samples were collected through incidental sampling after obtaining requisite permits from national authorities and in consultation with village leaders. No wildlife species were harmed or directly surveyed as part of this investigation.

Bushmeat samples (~ 250–500 g) were collected in double zip lock freezer bags containing sterile non-toxic Silica Gel desiccant moisture absorbers/dehumidifiers. The samples were stored in − 20 °C vehicle freezers prior to transferring to − 20 °C solar freezers in the field. The samples were then transported to the Nelson Mandela African Institution of Science and Technology (NM-AIST) in Arusha via vehicle freezers, where they were stored at − 80 °C until processing. Cold chain maintenance was ensured through temperature trackers included with the samples throughout the entire process. Sample identification sheets were completed for all collected samples and the metadata, including information regarding sample condition, wildlife species, the collection site (using GPS coordinates), and seasonality.

### Nucleic acid extraction

To avoid potential issues associated with surface contamination, three replicates of ~ 120 mg each, were obtained from the muscle tissue using sterile, disposable Rapid Core punch (World Precision Instruments, Sarasota, FL) and sterile disposable safety scalpels (VWR, Bridgeport, NJ). The samples were placed in the MagMAX™ Lysis/Binding Solution Concentrate buffer (Thermo Fisher Scientific, Waltham, Massachusetts). The homogenization protocol was optimized and the settings with the best DNA yield for both fresh and processed samples can be found in the Supplemental Appendix 1.

Nucleic acid extractions were performed using MagMAX™ 96 DNA Multi-Sample Kit (Thermo Fisher Scientific, Grand Island, NY) and KingFisher Flex automated DNA purification system (Thermo Fisher Scientific, Grand Island, NY) per manufacturer’s instructions with minor modifications. If the yield and purity of extractions were not adequate for a specific sample, such as the DNA concentration was less than 4 ng/µl and/or if the quality of the amplicons analyzed by the Bioanalyzer did not pass the quality control, DNA was extracted manually using DNeasy PowerSoil Kit (Qiagen, Hilden, Germany) per manufacturer’s instructions. Extracted DNA was quantified using Qubit™ 3.0 Fluorometer (Thermo Fisher scientific, Grand Island, NY). To ensure purified DNA was of high-quality, DNA was also visualized through agarose gel electrophoresis.

### Real time PCR

Amplification reactions for the detection of all pathogens (*B. anthracis*, *Brucella* and *Coxiella*) were carried out in 20 μl reactions with both positive and negative controls (Supplemental Table 1)^33^. Each PCR reaction included 10 μl of 2 × Taqman Fast Advanced master mix (Applied Biosystems, Woodward, Austin, USA), 2 μl of 5 uM of each forward and reverse primer (Supplemental Table 1), 1 μl of 5 μM probe (Supplemental Table 1), 1 μl of UltraPure™ DNase/RNase-Free distilled water (Thermo Fisher scientific, Grand Island, NY) and 4 μl of DNA template. Thermal cycling reactions were performed using 7500 Fast Real Time PCR System (Applied Biosystems, Waltham, MA). Targeted genes for *B. anthracis* included Protective antigen (PA) encoded by plasmid pX01 and Capsule B (CAPB2) encoded by plasmid pX02 (Supplemental Table 1). Samples were considered positive for *B. anthracis* when both plasmids, pXO1 and pXO2, were detected through RT-PCR. Outer Membrane Protein 2b (OMP2b) was the targeted gene to detect *Brucella* species and to detect *Coxiella burnetti*, Insertion element IS1111 was used as the target gene. Supplemental Table 1 contains the name of the target genes, sequences for forward and reverse primers and probes, and the PCR run condition. All positive samples were verified through repeating the nucleic acid extraction from the original sample and RT-PCRs were performed in technical triplicates.

**Table 1:** Wildlife species distribution, counts, and prevalence of pathogens in each regions of Serengeti, Ruaha, and Selous.

Serengeti				Ruaha				Selous			
Species	Count	Positive	Prevalence (95% CI)	Species	Total	Positive	Prevalence (95% CI)	Species	Total	Positive	Prevalence (95% CI)
Wildebeest	420	14	3.3 (1.9–5.7)	Dikdik	422	21	5.0 (3.2–7.6)	Gazelle	243	2	0.8 (0.14–3.3)
Buffalo	159	1	0.6 (0.3–4.0)	Gazelle	335	–	–	Kudu	109	–	–
Zebra	61	2	3.3 (0.6–12.4)	Kudu	171	–	–	Buffalo	89	2	2.2 (0.4–8.6)
Impala	60	1	1.7 (0.1–10.1)	Impala	108	1	0.93 (0.05–5.8)	Dikdik	81	1	1.2 (0.06–7.6)
Gazelle	26	–	–	Buffalo	95	1	1.1 (0.05–6.6)	Hartebeest	79	–	–
Bushpig	25	1	4 (2.1–22.3)	Hartebeest	95	–	–	Widebeest	65	1	1.5 (0.08–9.4)
Unknown	25	–	–	Bushpig	73	–	–	Bushpig	55	2	3.6 (0.6–13.6)
Topi	22	1	4.5 (0.2–24.9)	Griaffe	65	–	–	Impala	46	6	13.0 (5.4–26.9)
Eland	19	1	5.3 (0.3–28.1)	Duiker	55	–	–	Bushbuck	42	–	–
Giraffe	19	–	–	Reedbuck	37	–	–	Unknown	40	–	–
Ostrich	12	–	–	Warthog	34	–	–	Hippo	39	2	5.1 (0.9–18.6)
Warthog	11	–	–	Zebra	34	–	–	Hare	37	3	8 (2.1–23)
Leopard	8	–	–	Sable	28	–	–	Cattle	35	9	25.7 (13.1–45.7)
Porcupine	7	–	–	Unknown	20	–	–	Ostrich	33	–	–
Waterbuck	7	–	–	Wild cat	20	–	–	Zebra	33	3	9.1 (2.4–25.5)
Dikdik	6	–	–	Mongoose	18	–	–	Duiker	29	–	–
Hartebeest	6	–	–	Roan	13	–	–	Cane rat	28	1	3.6 (0.2–20.2)
Hippo	6	–	–	Hare	12	–	–	Eland	25	–	–
Steenbok	4	–	–	Elephant	11	–	–	Reedbuck	16	–	–
Antelope	1	–	–	Eland	8	–	–	Giraffe	15	–	–
Elephant	1	–	–	Bushbuck	7	–	–	Porcupine	11	–	–
Hare	1	–	–	Ostrich	5	–	–	Waterbuck	9	–	–
Hyrax	1	–	–	Porcupine	5	–	–	Elephant	8	–	–
Lion	1	–	–	Cane rat	4	–	–	Warthog	5	–	–
Sheep	1	–	–	Cattle	4	–	–	Sable	4	–	–
				Wildebeest	3	1	33.3 (NA)	Antelope	3	–	–
				Baboon	2	–	–	Galago	2	–	–
				Vervet monkey	2	–	–	Sheep	2	–	–
				Aardvark	1	–	–	Baboon	1	–	–
				Hippo	1	–	–	Mongoose	1	–	–
								Roan	1	–	–
								Suni	1	–	–
Total	909	21	2.3 (1.3–3.3)	Total	1688	24	1.4 (0.9–2.0)	Total	1187	32	2.7 (1.8–3.6)

"–": no data; NA: result not reliable and not included.

### Microbiome sequencing

The purified DNA was transported to the Biosciences eastern and central Africa-International Livestock Research Institute (BecA-ILRI) Hub in Nairobi, Kenya for the microbiome sequencing. The V3-V4 hypervariable region of the 16S rRNA gene was sequenced on the Illumina MiSeq System generating 2 × 300 bp paired-end reads (see methods in Supplemental Appendix 1). Sequence datasets are deposited in NCBI’s Sequence Read Archive (SRA) repository (Bioproject PRJNA477349).

### Bioinformatics for microbiota composition

To guarantee a high quality data set, microbiome analyses were performed using an open source platform, Empowering the Development of Genomics Expertise (EDGE)^34^. The EDGE Bioinformatics platform (v2.3.0) implementation is based on QIIME v1.9.1 and includes demultiplexing and quality filtering, operational taxonomic unit (OTU) clustering, taxonomic assignment, phylogenetic reconstruction, diversity analyses, and visualizations of each of these analyses^34^. QIIME started the pre-processing step with 43,095,486 paired-end reads (in FASTQ format) by removing reads with a PHRED quality score lower than Q20. Data were further filtered to remove reads with more than one ambiguous base. If less than 50% of a read contained consecutive high-quality bases, the read was discarded. The forward and reverse reads were merged before analysis. A 94% nucleotide identity sequence clustering threshold (approximately genus level) was used to generate OTUs. The representative sequence for each OTU was then queried against the Greengenes database^35^ for taxonomic classification assignment. The depth filter was set to 1000 reads—any barcoded sample that had less than 1000 assembled reads did not undergo taxonomic classification or further alpha- and beta-diversity analyses. Next, the integrated phylotype command generated the consensus taxonomy using phylotype-based approach, where taxonomy-linked sequences were assigned to OTUs based on similarity.

### Statistical analysis

All the statistical analyses were performed using the software R (v1.2.1335, RStudio, Inc), if not otherwise stated. The proportion of positive samples by season (dry, rainy), sampling region (Serengeti, Ruaha, Selous) and meat condition (fresh, processed) was estimated using *prop.test* with continuity correction function in the ‘stats’ package^36^. The relative risk of a sample being positive for one or more of the select pathogens was calculated using the risk-ratio function of the ‘epitools’ package^36^. To investigate the additive effects of all three categories on the outcome of a sample being positive for any of the pathogens, a multivariate analysis was performed using logistic Generalized Linear Model (GLM with logit link) with region, season and meat condition included as categorical variables^37^. To examine the potential for sample size biases in the microbiota sequencing, a rarefaction analysis was conducted where the fraction of taxa was captured for each sample^38^. Results show that we were able to consistently identify all the detectable OTUs present in every sample with a minimum reading depth of 14,050 sequences per sample. This suggests that the variation of the microbiota among samples is not likely caused by low sequence coverage and that further sequencing would not detect additional genera (Supplemental Figure 1). The microbiome alpha diversity (species richness) was calculated using the Shannon Diversity Index in the package ‘Vegan’^39^. Statistical analysis for the microbial diversity between the groups was performed using Welch’s *t*-test^37^. Hierarchical clustering of the samples based on relative abundance of phyla was performed using the package ‘pvclust’, with 1000 bootstrapped replications of the clusters^40^. To examine the beta diversity of the samples, Principal Coordinate Analysis (PCoA) was performed using the Bray–Curtis Matrix at the phylum level using the package ‘Vegan’^39^. Groups were clustered graphing ellipses estimating the 95% confidence interval (CI) around the average value, for each variable.

**Figure 1:**
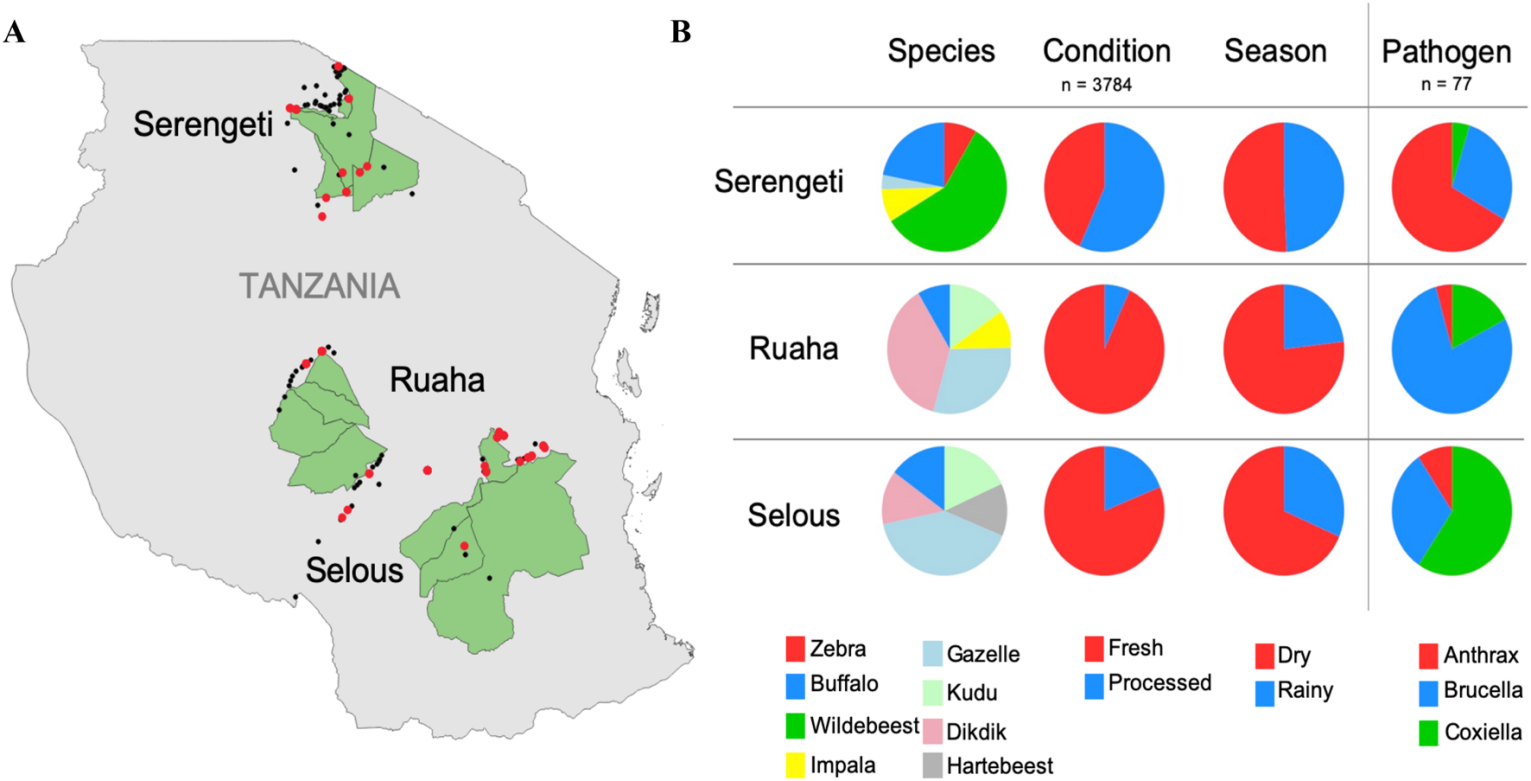
Map and distribution of bushmeat samples collected from the three ecosystems in Tanzania. (**A**) Map of the sample collection sites. In the map, the three regions are shown in green. The points show the distribution of samples collected (black circles) and samples positive for one of the three pathogens (red circles). (**B**) Distribution of the collected bushmeat samples. The pie charts show the stratification of the collected bushmeat samples. For the species category (*n* = 3784), the top 5 most abundant species collected from the particular region are shown: Serengeti–Wildebeest (green), Buffalo (blue), Zebra (red), Impala (yellow), Gazelle (light blue); Ruaha–Dik-dik (pink), Gazelle (light blue), Kudu (light green), Impala (yellow), Buffalo (blue); Selous–Gazelle (light blue), Buffalo (blue), Dik-dik (pink), Kudu (light green), Hartebeest (grey). For the condition category (*n* = 3784), the red represents fresh samples and blue represents processed samples. For the season category (*n* = 3784), dry and rainy seasons are represented by the red and blue colors, respectively. The pathogen column (*n* = 77) shows the proportion of each pathogen from each region with *B. anthracis* shown in red, *Brucella* in blue, and *Coxiella* in green. The samples were collected from both dry and rainy seasons, Ruaha (dry; I = 1297; rainy = 391), Selous (dry = 813; rainy = 374), Serengeti (dry = 461; rainy = 448), and from two sample conditions, Ruaha (fresh = 1571; processed = 117), Selous (fresh = 962; processed = 223), Serengeti (fresh = 393; processed = 516). The map was generated using the QGIS Geographic Information System software. "QGIS Development Team (2019). Open- Source Geospatial Foundation Project. Version 3.4.9-Madeira. http://qgis.osgeo.org".

## Results

### Bushmeat samples

A total of 3784 samples were collected in and around the three geographical study locations: Ruaha (*n* = 1688; 33 villages), Selous (*n* = 1187; 27 villages), and Serengeti (*n* = 909; 54 villages) as shown in Fig. 1A. The samples represent wildlife from both high and low densities and include the predominant herbivores, such as wildebeest (*Connochaetes taurinus*), zebra (*Equus burchellii quagga*), buffalo (*Syncerus caffer*), impala (*Aepyceros melampus*), dik-dik (*Madoqua* spp.), and other less common species, including eland (*Tragelaphus oryx*), topi (*Damaliscus lunatus*), and the African savannah hare (*Lepus microtis*) (Table 1).

### Signatures of select agents in bushmeat samples

**Figure 2:**
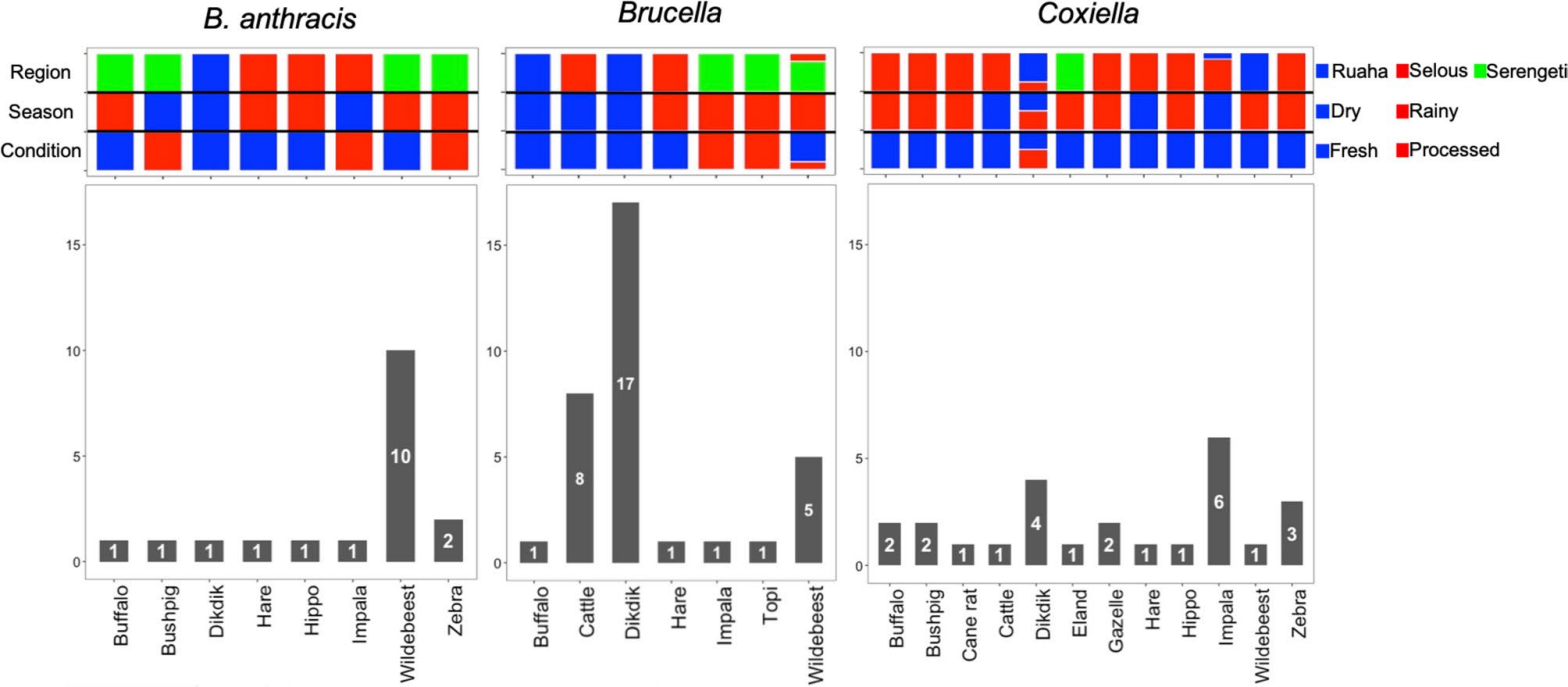
Counts of Bushmeat Samples Positive for Select Agents. The count of positives for each species are included in the bar charts (with the total count in the bar with white font), separated by *B. anthracis*-positive in the left panel, *Brucella*-positive in the middle panel, and *Coxiella*-positive in the right panel. The coloring above the bar graphs show the proportion of samples within the species that are in each category (Region, Season, and Condition). The blue color represents samples collected from Ruaha, collected during dry season, and fresh samples. The red color represents samples collected from Selous, collected during Rainy season, and processed samples. The green color represents samples collected from the Serengeti. For instance, in the *B. anthracis* panel, one positive fresh (blue) buffalo sample was collected during rainy season (red), from Serengeti region (green).

Of the 3784 collected bushmeat samples, 2.03% (*n* = 77, CI 1.6–2.5%) samples were identified as positive for at least one of the three tested pathogens, *B. anthracis*, *Brucella*, and *Coxiella*. There was an overall prevalence of 0.48% (*n* = 18, CI 0.3–0.8%) of *B. anthracis* in the collected bushmeat samples, 0.9% (*n* = 34, CI 0.6–1.3%) prevalence of *Brucella*, and 0.7% (*n* = 25, CI 0.4–1.0%) prevalence of *Coxiella* (Fig. 2). The proportion of the positive samples from each location indicates that the highest prevalence of *B. anthracis* was found in the Serengeti at 78% (*n* = 14, CI 0.6–1.0%), the highest percentage of *Brucella* in Ruaha at 53% (*n* = 18, CI 0.4–0.7%), and the highest percentage of *Coxiella* was found in Selous at 76% (*n* = 19, CI 0.6–1.0%) (Fig. 1B, Supplemental Table 2). Overall, from each location, there was a 2.3% (*n* = 21, CI 1.3–3.3%) prevalence of the three select agents in the Serengeti, 1.4% (*n* = 24, CI 0.9–2.0%) prevalence in Ruaha, and 2.7% (*n* = 32, CI 1.8–3.6%) prevalence in Selous (Table 1). All Confidence Intervals (CI) were calculated at 95% threshold.

**Table 2:** Multi-variate analysis of the variables associated with the positive bushmeat samples.

Variable	Log odds	SE	*z*-value	*p*-value
Season-rainy	0.6908	0.242	2.854	0.00431
Condition-processed	− 1.107	0.3744	− 2.957	0.00311
Region-Selous	0.6869	0.2748	2.499	0.01244
Region-Serengeti	0.6769	0.3291	2.057	0.03973

Samples collected from rainy season increase the log odds of being positive by 0.69. Processed samples decrease the log odds of being positive by 1.11. Samples collected from Selous and Serengeti increase the log odds of being positive by 0.69 AND by 0.68, respectively.

The samples positive for *B. anthracis* were primarily wildebeest (*n* = 10) and the samples positive for *Brucella* were mostly from dik-dik (*n* = 17) (Fig. 2). The samples positive for *Coxiella* had a larger range of species, with impala and dik-dik being the most prevalent (Fig. 2). Remarkably, all of the wildebeest samples that were positive for *B. anthracis* were fresh samples from the Serengeti collected during the rainy season (Fig. 2). Also, all of the dik-dik samples positive for *Brucella* were fresh samples from Ruaha collected during the dry season (Fig. 2). Only one of the *Coxiella*-positive samples was from the Serengeti and this was a fresh sample from an eland collected during rainy season (Fig. 2).

### Relative risk and multivariate analyses

**Figure 3:**
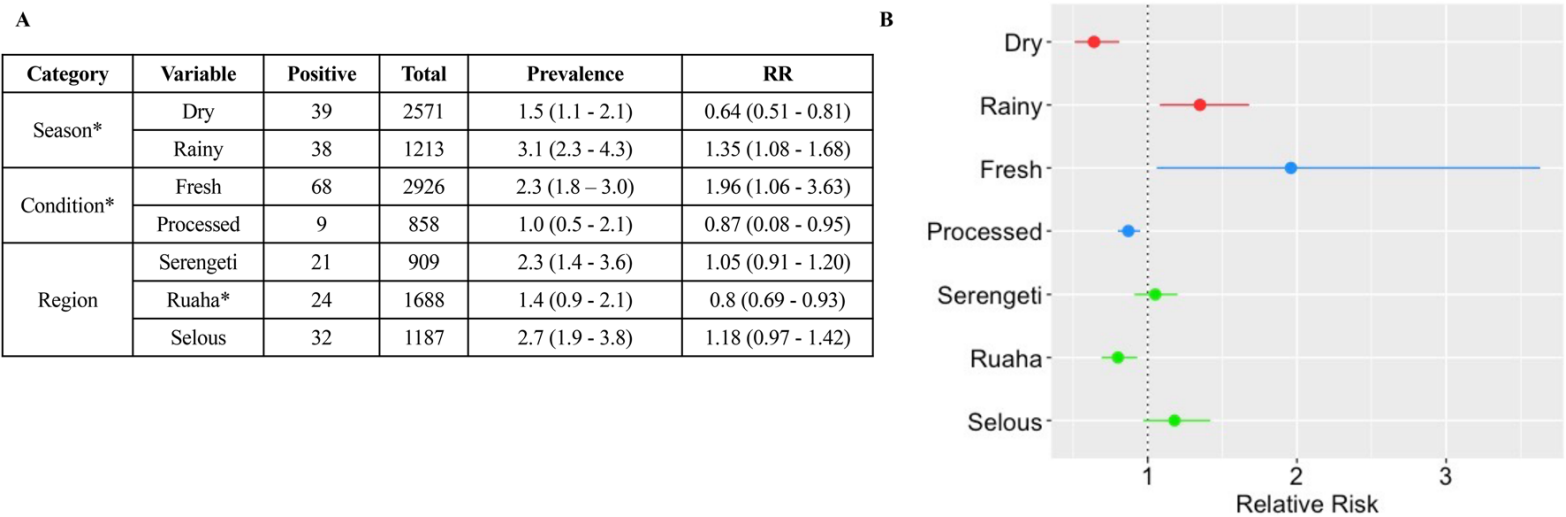
Relative risk (RR) of the positive bushmeat samples associated with different variables. (**A**) Counts (positive and total samples), prevalence and RR associated with each variable. The 95% confidence intervals are reported in parethesis. (**B**) Graph of the RR for each variable. The relative risk (pointS) and 95% confidence interval (lines) for each variable are reported for the seasonal data (red), sample condition (blue) and regions (green). Results indicate that there is a 71% higher risk of a sample collected from rainy season being positive than samples collected during dry season. There is a 109% higher risk of a fresh sample being positive than processed sample. There is also a 64% lower risk of a sample collected from Ruaha being positive that the other regions (the RR of samples collected from both Selous and Serengeti is 1.44). Significant differences in RR analysis are reported (*p < 0.05).

The relative risk (RR) associated with a sample being positive for one or more of the select pathogens was determined (Fig. 3). The relative risk for pathogens collected during rainy season was 1.4 (CI 1.1–1.7) and significantly higher compared to the RR of 0.64 (CI 0.5–0.8) for samples collected during dry season; in other words, there was a 71% higher risk of samples collected during rainy season to be positive. The relative risk of getting a pathogen from fresh samples was 1.96 (CI 1.1–3.6) and significantly higher than the relative risk of 0.87 (CI 0.08–0.95) for processed samples, indicating a 109% higher risk for a fresh sample being found to be positive than those that were processed. The RR of samples from Ruaha was 0.8 (CI 0.7–0.9) and it was significantly lower than samples collected from the other locations, in particular, Selous (RR = 1.2; CI 0.97–1.4) (Fig. 3).

Multivariate analysis was undertaken to investigate the combined effects of all three categories on the outcome of a sample being positive for any of the pathogens. Results showed that samples collected during the rainy seasons increase the log odds of being positive by 0.70, compared to the dry season. On the contrary, processed samples decrease the log odds of being positive by 1.1, compared to fresh samples. Among the regions, analysis indicated that compared to Ruaha, samples collected from Selous increase the log odds of being positive by 0.70, and samples collected from Serengeti increase the log odds of being positive by 0.70 (Table 2).

### The microbiome associated with select pathogen-positive bushmeat samples

**Figure 4:**
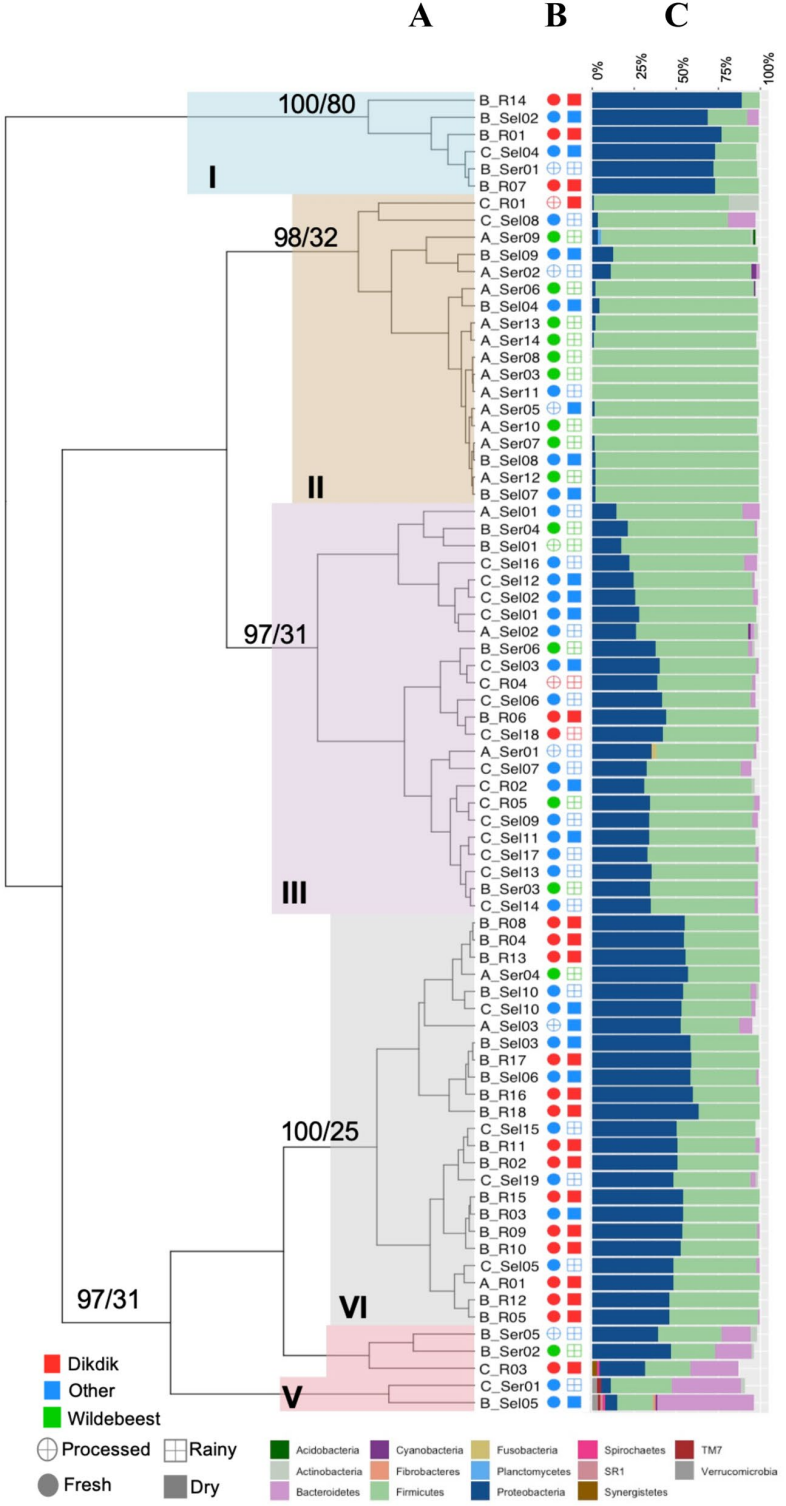
Hierarchical clustering of the samples positive for one of the three pathogens based on the relative abundance of phyla in each sample. (**A**) Hierarchical clustering of samples. The samples grouped into 5 clusters: I—blue, II—tan, III—purple, IV—grey, V—red. The samples are labeled with the first letter representing the pathogen (A—*B. anthracis*, B—*Brucella*, C—*Coxiella*) and the ending representing the ecosystem (R—Ruaha, Sel—Selous, Ser—Serengeti). The AU/BP values for the clustering are shown at the base of each node. (**B**) Sample metadata. These columns show the other metadata associated with each sample. The open circles represent processed samples, and the closed are fresh samples. The open squares are from rainy season and the closed squares are from dry season. The shapes are colored according to species of wildlife from which the bushmeat originated: dik-dik—red, wildebeest—green, all other species—blue. (**C**) Bar graph of the relative abundance of phyla. The relative abundance of phyla > 1% is shown in the stacked bar graph. The colors corresponding to each respective phylum is in the legend at the bottom.

Microbiome characterization of the 77 positive samples showed that the two most abundant phyla across all samples were *Firmicutes* and *Proteobacteria* (Fig. 4) (Supplemental Table 2). Rarefaction analyses showed consistent identification of the detectable OTUs present in each sample with a minimum reading depth of 14,050 sequences per sample^38^. This suggests that the variation of the microbiota among samples is not likely caused by low sequence coverage (Supplemental Figure 1). The alpha and beta diversity for the different variables associated with each sample was assessed. The first two principal coordinates of the Principal Coordinate Analysis (PCoA) accounted for 95% of the total variation. The first principal coordinate was driven by the effect of the relative abundance of *Proteobacteria* (coefficient = 0.86) and *Firmicutes* (− 0.90) and the second principal coordinate by the relative abundance of *Bacteroidetes* (0.99) (Supplemental Figure 2).

In addition, lower alpha diversity and significant differences in beta diversity were observed in *B. anthracis*-positive samples, and samples collected from the Serengeti, which were primarily wildebeest. The results showed that *B. anthracis*-positive samples had significantly lower alpha diversity than either of the *Brucella*- or *Coxiella*-positive samples (*p*-value < 0.001; Supplemental Figure 3) and tended to cluster separately in the Principal Coordinate Analysis (PCoA) (Supplemental Figure 3). There was a similar pattern for wildebeest samples, which showed lower overall diversity and clustering separately from dik-dik or other species (*p*-value < 0.001; Supplemental Figures 2 and 3).

Hierarchical clustering based on the relative abundance of phyla grouped the samples into five distinct clusters (Fig. 4A). The associated Approximately Unbiased (AU) and Bootstrap Probability (BP) are represented at the base node of each cluster (AU > 95 are statistically significant). Cluster I (*n* = 6) contained six samples, of which five were *Brucella*-positive with the majority of the samples being freshly collected (Fig. 4). Cluster II (*n* = 18) included 12 *B. anthracis*-positive samples collected from Serengeti, of which eight were from fresh wildebeest samples collected during the rainy season. Cluster III (*n* = 24) included 16 *Coxiella*-positive samples, of which 15 were fresh, with thirteen of these collected from the Selous ecosystem. Samples in cluster IV (*n* = 24) included 17 *Brucella*-positive samples collected from Ruaha and Selous, four *Coxiella*-positive samples collected from Selous, and three *B. anthracis*-positive samples collected from the Serengeti ecosystem. The final cluster, cluster V (*n* = 5) was the most diverse and included three *Brucella*-positive and two *Coxiella*-positive samples (Fig. 4).

The relative abundance was also analyzed at the family level (Supplemental Figure 4). At this level, the top three most abundant taxa in these samples include, *Streptococcaceae* (26%), *Enterobacteriaceae* (22%)*,* and *Moraxellaceae* (14%). The alpha diversity revealed differences according to pathogen, host species, and region (Supplemental Figure 5) and the beta diversity clustered the wildebeest samples separately from the other species (Supplemental Figure 6).

**Figure 5:**
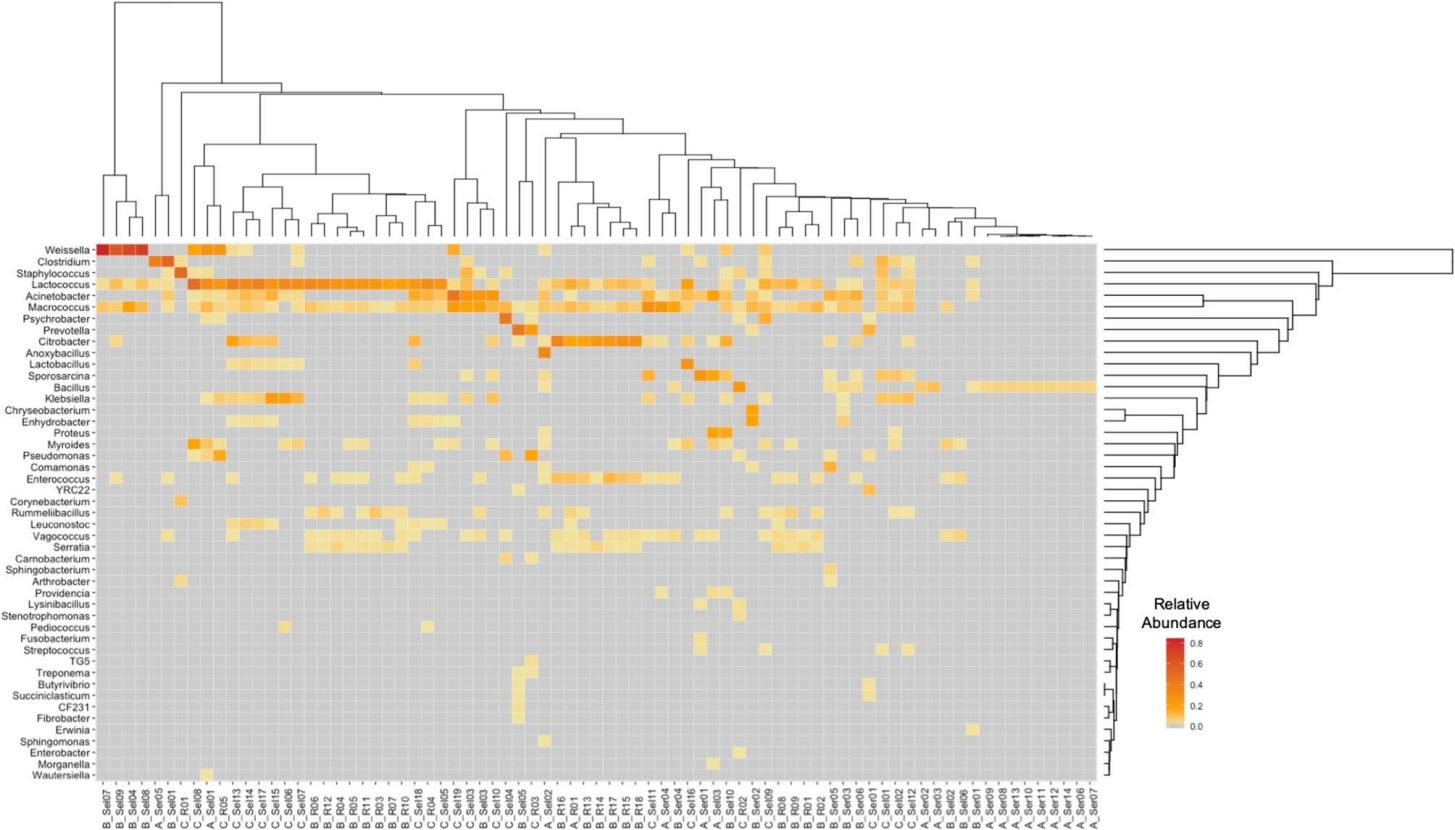
Heatmap of the genus-level relative abundance in the samples positive for one of the three pathogens. The heatmap shows the relative abundance for any genus present at > 1%. The color ranges from pale yellow (low abundance) to dark orange (high abundance). Hierarchical clustering was performed for both the samples and the genera. The samples are labeled with the first letter representing the pathogen (A—*B. anthracis*, B—*Brucella*, C—*Coxiella*) and the ending representing the regions (R—Ruaha, Sel—Selous, Ser—Serengeti).

Interestingly, when the data were investigated at the genus-level, hierarchical clustering tended to group the samples by pathogen. The *B. anthracis*-positive samples were high in *Bacillus*, which was not found in the *Brucella*- or *Coxiella*-positive samples (Fig. 5). On the other hand, the *Brucella*- and *Coxiella*-positive samples showed a higher abundance of species represented by the *Lactococcus* and *Macrococcus* genera (Fig. 5). These differences were further confirmed through examining the alpha and beta diversity associated with the samples. Within each variable, samples that had lower alpha diversity were *B. anthracis*-positive, wildebeest samples, and those collected from Serengeti (Supplemental Figure 7), which were also shown to be the most significant in the PCoA analysis (Supplemental Figure 8).

## Discussion

A goal of this study was to assess the potential for risk of human exposure to three select agents, *Bacillus anthracis*, *Brucella* spp., and *Coxiella burnetii* associated with bushmeat handling and consumption in Tanzania. Our initial focus was on three major locations in Tanzania: Ruaha and Selous in the south and the Serengeti in the north. Sites were selected to represent the distinct environments (habitat and climate) with diverse wildlife composition and density, and proximity to major urban centers (including Arusha, Dodoma and Dar es Salaam) where the bushmeat is often sold.

While the presence of DNA signatures does not imply the presence of a viable organism, given that bushmeat consumption is as high as 5 meals per week in some households, and with an overall pathogen prevalence of 2.03% (1.6–2.5%), this risk of exposure remains a concern and worthy of further investigation. This is especially problematic considering that the risks associated with endemic infections such as *Brucella* and *Coxiella* often go unrecognized and remain under-reported despite the considerable risks of causing disease^41^.

Relative risk associated with positive DNA signatures was assessed for variables including season, sample condition, animal species, and regions (ecosystems) and revealed notable associations. There was a higher risk of a sample being positive if it was collected during a rainy season, or if the sample was fresh, in contrast there was a lower risk if the sample was collected from Ruaha, than Serengeti or Selous (Fig. 3). Studies have shown that climate-related factors, such as prolonged rain, can contribute to disease outbreaks^42^. Changes in weather conditions force the movement and interactions of animals where rainy seasons and/or droughts affect animals spatial grazing patterns^43,44^. Also, exposure to anthrax may be higher for migratory ungulates like wildebeest that are found in different parts of the regions throughout the year^3^. This may be one reason why there is a higher likelihood of positive samples being collected during the rainy season. Also, during the rainy season there may be a higher consumption of fresh samples, which is consistent with the higher risk of positives in fresh samples, probably associated with methods of processing being unavailable or unsuitable during this season, or simply a higher availability of fresh meat. High salt, UV exposure, and boiling of processed samples may reduce pathogen loads associated with the fresh meat samples^45,46^; however, UV light does not necessarily decrease the microbial content of meat^47,48^. Results also indicate that there is a lower risk of a sample being positive if it was collected from Ruaha. There are some substantial differences among the three ecosystems, both biotic and abiotic, and we have no evidence of a dominant factor affecting the samples, and suggest that multiple components might be contributing to the relative risks associated with each ecosystem^49,50^.

Findings indicated that most of the differences in the microbiome are driven by the relative abundance of *Firmicutes* and *Proteobacteria*, as demonstrated by the PCoA (Supplemental Figure 2). This is expected, since *B. anthracis* belongs to the *Firmicutes* phylum and *Brucella* and *Coxiella* both belong to *Proteobacteria*. The microbiome analysis revealed significant lower alpha diversity and PCoA clustering associated with the *B. anthracis*-positive samples, wildebeest samples, and samples collected from the Serengeti (Supplemental Figures 2, 3, 5, 6, 7, and 8). These results are also supported by the hierarchical clustering at both the phylum- and the genus-levels, where the *B. anthracis*-positive samples tended to cluster together (Fig. 4–Cluster II and Fig. 5). This is expected since the highest abundance of *B. anthracis*-positive samples were in the Serengeti, where most of the wildebeest samples were collected (Fig. 1). These results are consistent with previous studies that documented many Anthrax outbreaks in Northern Tanzania, and particularly in the Serengeti^8,11,21,51^.

Samples that were collected in the same condition were generally available to be sold to the public, hence they represent microbial communities that are endogenous to the samples, as well as the microbiota that is acquired during harvesting, processing, and transporting. While the analysis suggests that fresh samples, samples collected during rainy season, and samples collected from the Serengeti and Selous had a higher likelihood of being positive for one of the three pathogens, positive samples carrying DNA signatures of the tested select agents were also collected during dry season, from other animal species, and ecosystems. Interestingly, the results also revealed that a subset (*n* = 39) of purported “bushmeat” samples from Selous that were instead speciated as being of cattle in origin had a combined prevalence of 28.7% of the three select agents, highlighting a potential public health risk associated with the endemic diseases in livestock production. This suggests that *B. anthracis*, *Brucella,* and *Coxiella* circulate throughout the year in all the ecosystems and confirms their endemicity and the recurrent risk for the nearby communities, including the larger markets where the meat is regularly sold.

While this study represents the first comprehensive assessments of the prevalence of DNA signatures of select agents in bushmeat in Tanzania, there are several unanswered questions that remain. For instance, it remains unclear how these DNA signatures represent / correlate with presence of viable pathogens and, with this, the risk of transmission to humans. It is also unclear whether there are any differences in the health risks associated with exposure during harvest, butchering, handling or consuming of bushmeat. Future studies investigating the role of other select agents (bacterial and viral) in the ecosystems, and the role of endemic and emerging strains in the microbial community, caused by sedentary and migrating animal species in the region are also warranted since the extent of an outbreak varies from year to year and place to place. In conclusion, the current investigations provide important insights and a rational basis for the formulation of future studies to assess and address the public health and emerging infectious disease risks associated with bushmeat harvesting, trade, and consumption in Tanzania and elsewhere.

## Supplementary Information

Supplementary Information 1.

Supplementary Information 2.

Supplementary Information 3.

Supplementary Information 4.

## Data Availability

The datasets generated and analyzed during the current study are available in the NCBI Sequence Read Archive (SRA) repository, https://trace.ncbi.nlm.nih.gov/Traces/sra/?study=SRP151593.
