# Supplementary material for: Identification of Bacillus anthracis, Brucella spp., and Coxiella burnetii DNA signatures from bushmeat
Source: Sci Rep. 2021 Jul 21;11:14876. doi: 10.1038/s41598-021-94112-9 (PMC8295346; doi:10.1038/s41598-021-94112-9)
Supplement: Supplementary file 1 — Supplementary Information 1. [file 41598_2021_94112_MOESM1_ESM.pdf]

Appendix 1

IDENTIFICATION OF *BACILLUS ANTHRACIS, BRUCELLA SPP.*, AND *COXIELLA BURNETII* DNA SIGNATURES FROM BUSHMEAT

Robab Katani[1,2†], Megan A. Schilling[2,3†], Beatus Lyimo[4†], Ernest Eblate[4,5], Andimile Martin[4], Triza Tonui[6], Isabella M. Cattadori[2,4,7], Stephen C. Francesconi[8], Anna B. Estes[2,4**], Dennis Rentsch[9], Sreenidhi Srinivasan[2], Samson Lyimo[4], Lidia Munuo[4], Christian K. Tiambo[6], Francesca Stomeo[6***], Paul Gwakisa[10], Fausta Mosha[11], Peter J. Hudson[1,2,4,7], Joram J. Buza[4] and Vivek Kapur[1,2,3,4*]

[1]Applied Biological and Biosecurity Research Laboratory, Pennsylvania State University, University Park, Pennsylvania, USA

[2]The Huck Institutes of the Life Sciences, Pennsylvania State University, University Park, Pennsylvania, USA

[3]Department of Animal Science, Pennsylvania State University, University Park, Pennsylvania, USA

[4]Nelson Mandela African Institution of Science and Technology, Arusha, Tanzania

[5]Tanzania Wildlife Research Institute, Arusha, Tanzania

[6]Biosciences eastern and central Africa-International Livestock Research Institute (BecA-ILRI) Hub, Nairobi, Kenya

[7]Department of Biology, Pennsylvania State University, University Park

[8] Naval Medical Research Center, Fort Detrick, MD, USA

[9]Lincoln Park Zoo, Chicago, Illinois, USA

[10] Sokoine University of Agriculture, Morogoro, Tanzania

[11]Ministry of Health Community Development Gender Elderly and Children, Dar es Salaam Tanzania

*Corresponding author: Vivek Kapur, vkapur@psu.edu

† Contributed equally to this work.

****** Currently: Dept. of Environmental Studies, Carleton College, Northfield, MN, USA

*** Currently: The European Molecular Biology Laboratory (EMBL), Heidelberg, German

Appendix 1

DNA extraction:

The homogenization protocol was optimized and the settings with the best DNA yield for both fresh and process samples were used. In brief, fresh samples were processed for 45 second at 5.5 m/s using a bead beater, Bead Ruptor 24 Bead Mill Homogenizer (Omni International, Kennesaw, GA), and 2.3 MM zirconia beads (BioSpec Products, Bartlesville, OK) in the MagMAX™ Lysis/Binding buffer. Processed samples were pre-soaked in the UltraPure DNase/RNase-Free Distilled Water (Thermo Fisher Scientific, Grand Island, NY) at 4°C overnight, prior to homogenization. The homogenization was conducted for three 30 second intervals at 5.5 m/s with 2.3 MM zirconia beads in the MagMAX™ Lysis/Binding buffer.

Microbiome Sequencing

The V3-V4 hypervariable region of the 16S rRNA gene (~ 460bp) was amplified using the Illumina 16S rRNA primers for the V3-V4 region prior to sequencing. The full-length primer sequences, to follow the protocol targeting this region are: 16SPCR1_Forward Primer = 5'TCG TCG GCA GCG TCA GAT GTG TAT AAG AGA CAG CCT ACG GGN GGC WGC AG and 16SPCR_Reverse Primer = 5'GTC TCG TGG GCT CGG AGA TGT GTA TAA GAG ACA GGA CTA CHV GGG TAT CTA ATC C [36].

Appendix 1

The PCR was performed using 12.5 µl 2X KAPA HiFi HotStart Ready Mix Kit (KAPA Biosystems, Wilmington, MA). The premix was complemented with 5 µl each of 1 µM forward and reverse primers (Bioneer, Alameda, CA) 10 µl Nuclease-free water (Ambion, Life Technologies, Waltham, MA) and 2.5µl of genomic DNA in a final volume of 25 µl. The PCR reaction was carried out on a Thermal Cycler (Life Technologies, Carlsbad, CA) as follows: an initial denaturation step (95°C for 3 min), 25 cycles of amplification (95°C for 30sec, 55°C for 30sec and 72°C for 30sec) and a final elongation step at 72°C for 5 min. Amplicons were then purified using the QIAquick PCR purification kit (Qiagen, USA) following the manufacturer's instructions. Using Qubit dsDNA BR Assay kit (ThermoFisher Scientific, Waltham, MA), the concentration of the purified amplicons was determined.

During the sequencing step, sample multiplexing was performed using a unique reverse primer per sample [37]. The quality of a set of amplicons (per sequencing run) was tested using Agilent DNA 7500 chips using the Bioanalyzer 2100 (Agilent Technologies, Santa Clara, CA, USA). 40 ng of each sample library was pooled in order to normalize the libraries. The final pool was denatured using 0.2N NaOH and diluted to 12 pM. The PhiX Control v3 at 12 pM was added to the pool at 15% of the final volume as described in the Illumina procedure for sequencing of low diversity libraries. 600 µl of this treated pool mixture was loaded onto the Illumina MiSeq cartridge and sequenced on the Illumina MiSeq System generating 2x300 bp Paired-End Reads.
