# Supplementary material for: Identification of Bacillus anthracis, Brucella spp., and Coxiella burnetii DNA signatures from bushmeat
Source: Sci Rep. 2021 Jul 21;11:14876. doi: 10.1038/s41598-021-94112-9 (PMC8295346; doi:10.1038/s41598-021-94112-9)
Supplement: Supplementary file 2 — Supplementary Information 2. [file 41598_2021_94112_MOESM2_ESM.pdf]

IDENTIFICATION OF *BACILLUS ANTHRACIS*, *BRUCELLA SPP.*, AND *COXIELLA BURNETII* DNA SIGNATURES FROM BUSHMEAT

Robab Katani[1,2†], Megan A. Schilling[2,3†], Beatus Lyimo[4†], Ernest Eblate[4,5], Andimile Martin[4], Triza Tonui[6], Isabella M. Cattadori[2,4,7], Stephen C. Francesconi[8], Anna B. Estes[2,4**], Dennis Rentsch[9], Sreenidhi Srinivasan[2], Samson Lyimo[4], Lidia Munuo[4], Christian K. Tiambo[6], Francesca Stomeo[6***], Paul Gwakisa[10], Fausta Mosha[11], Peter J. Hudson[1,2,4,7], Joram J. Buza[4] and Vivek Kapur[1,2,3,4*]

Supplemental Table 1. List and sequences of primers and probes for pathogen detection.

Target	name	Nucleotide sequence (5' -3')		Probe [6FAM]	Quenchers	PCR conditions
		Forward	Reverse			
Bacillus anthracis	***Protective Antigen (PA) p* XO1**	TTCAAGTTGTACT GGACCGATTCTC	TCCATCATTGTC ACGGTCTGG	CCGTAGGTCCAGCAC TTGTACTTCGCTT	TAMRA	95ºC for 2 min, followed by 45 cycles of 95ºC for 10 sec and 60ºC for 20 sec
	***Capsule B (CAPB2) p* XO2**	GCTGACCAATCTA AGCCTGC	GGCAAAACATCC CTAGCAAA	TTGTAATTATGAATT GCCGCCCTGACC		
Brucella sp.	**Outer Membrane Protein 2b (OMP2b)**	ATGAACATCAAGA GCCTTCTCCTT	GGTGCCCGGAAT GTAGAAGTAG	TCTGGTTGCAGCTTC	NFQ-MGB	95ºC for 2 min followed by 45 cycles of 95ºC for 10 sec and 60ºC for 30 sec
Coxiella burnetti	**Insertion Element IS1111**	AATTTCATCGTTC CCGGCAG	GCCGCGTTTACT AATCCCCA	TGTCGGCGTTTATTG GGTTGGTCCC	TAMRA	96ºC for 2 min, followed by 45 cycles of 96ºC for 5 sec and 60ºC for 1 min
