# Supplementary material for: Identification of Bacillus anthracis, Brucella spp., and Coxiella burnetii DNA signatures from bushmeat
Source: Sci Rep. 2021 Jul 21;11:14876. doi: 10.1038/s41598-021-94112-9 (PMC8295346; doi:10.1038/s41598-021-94112-9)
Supplement: Supplementary file 3 — Supplementary Information 3. [file 41598_2021_94112_MOESM3_ESM.pdf]

IDENTIFICATION OF *BACILLUS ANTHRACIS* , *BRUCELLA SPP.* , AND *COXIELLA BURNETII* D

Robab Katani[1,2†], Megan A. Schilling[2,3†], Beatus Lyimo[4†], Ernest Eblate[4,5], Andimile Martin[4], Triza Tonui[6], Isab
Cattadori[2,4,7], Stephen C. Francesconi[8], Anna B. Estes[2,4**], Dennis Rentsch[9], Sreenidhi Srinivasan[2], Samson Lyi
Munuo[4], Christian K. Tiambo[6], Francesca Stomeo[6***], Paul Gwakisa[10], Fausta Mosha[11], Peter J. Hudson[1,2,4,7], J
Buza[4] and Vivek Kapur[1,2,3,4*]

Supplemental Table 2. Metadata for 77 bushmeat samples identified by real-time PCR as pathogen-positive.

Description	Species	Season	Condition	Region	Pathogen
A_R01	Dikdik	Dry	Fresh	Ruaha	Anthrax
A_Sel01	Hare	Rainy	Fresh	Selous	Anthrax
A_Sel02	Hippo	Rainy	Fresh	Selous	Anthrax
A_Ser06	Wildebeest	Rainy	Fresh	Serengeti	Anthrax
A_Ser07	Wildebeest	Rainy	Fresh	Serengeti	Anthrax
A_Ser08	Wildebeest	Rainy	Fresh	Serengeti	Anthrax
A_Ser03	Wildebeest	Rainy	Fresh	Serengeti	Anthrax
A_Ser09	Wildebeest	Rainy	Fresh	Serengeti	Anthrax
A_Ser10	Wildebeest	Rainy	Fresh	Serengeti	Anthrax
A_Ser11	Buffalo	Rainy	Fresh	Serengeti	Anthrax
A_Ser12	Wildebeest	Rainy	Fresh	Serengeti	Anthrax
A_Ser13	Wildebeest	Rainy	Fresh	Serengeti	Anthrax
A_Ser14	Wildebeest	Rainy	Fresh	Serengeti	Anthrax
A_Ser04	Wildebeest	Rainy	Fresh	Serengeti	Anthrax
B_R01	Dikdik	Dry	Fresh	Ruaha	Brucella
B_R02	Dikdik	Dry	Fresh	Ruaha	Brucella
B_R13	Dikdik	Dry	Fresh	Ruaha	Brucella
B_R03	Buffalo	Dry	Fresh	Ruaha	Brucella
B_R04	Dikdik	Dry	Fresh	Ruaha	Brucella
B_R05	Dikdik	Dry	Fresh	Ruaha	Brucella
B_R14	Dikdik	Dry	Fresh	Ruaha	Brucella
B_R06	Dikdik	Dry	Fresh	Ruaha	Brucella
B_R07	Dikdik	Dry	Fresh	Ruaha	Brucella
B_R08	Dikdik	Dry	Fresh	Ruaha	Brucella
B_R15	Dikdik	Dry	Fresh	Ruaha	Brucella
B_R16	Dikdik	Dry	Fresh	Ruaha	Brucella
B_R17	Dikdik	Dry	Fresh	Ruaha	Brucella
B_R09	Dikdik	Dry	Fresh	Ruaha	Brucella
B_R10	Dikdik	Dry	Fresh	Ruaha	Brucella
B_R11	Dikdik	Dry	Fresh	Ruaha	Brucella
B_R18	Dikdik	Dry	Fresh	Ruaha	Brucella
B_R12	Dikdik	Dry	Fresh	Ruaha	Brucella
B_Sel05	Cattle	Dry	Fresh	Selous	Brucella
B_Sel06	Cattle	Dry	Fresh	Selous	Brucella

B_Sel02	Cattle	Dry	Fresh	Selous	Brucella
B_Sel03	Cattle	Dry	Fresh	Selous	Brucella
B_Sel07	Cattle	Dry	Fresh	Selous	Brucella
B_Sel08	Cattle	Dry	Fresh	Selous	Brucella
B_Sel09	Cattle	Dry	Fresh	Selous	Brucella
B_Sel04	Cattle	Dry	Fresh	Selous	Brucella
B_Sel10	Hare	Rainy	Fresh	Selous	Brucella
B_Ser02	Wildebeest	Rainy	Fresh	Serengeti	Brucella
B_Ser03	Wildebeest	Rainy	Fresh	Serengeti	Brucella
B_Ser04	Wildebeest	Rainy	Fresh	Serengeti	Brucella
B_Ser06	Wildebeest	Rainy	Fresh	Serengeti	Brucella
C_R02	Impala	Dry	Fresh	Ruaha	Coxiella
C_R03	Dikdik	Dry	Fresh	Ruaha	Coxiella
C_R05	Wildebeest	Rainy	Fresh	Ruaha	Coxiella
C_Sel10	Hare	Dry	Fresh	Selous	Coxiella
C_Sel01	Impala	Dry	Fresh	Selous	Coxiella
C_Sel11	Impala	Dry	Fresh	Selous	Coxiella
C_Sel12	Impala	Dry	Fresh	Selous	Coxiella
C_Sel02	Impala	Dry	Fresh	Selous	Coxiella
C_Sel03	Impala	Dry	Fresh	Selous	Coxiella
C_Sel04	Cattle	Dry	Fresh	Selous	Coxiella
C_Sel05	Zebra	Rainy	Fresh	Selous	Coxiella
C_Sel13	Hippo	Rainy	Fresh	Selous	Coxiella
C_Sel14	Zebra	Rainy	Fresh	Selous	Coxiella
C_Sel15	Pig	Rainy	Fresh	Selous	Coxiella
C_Sel16	Buffalo	Rainy	Fresh	Selous	Coxiella
C_Sel17	Zebra	Rainy	Fresh	Selous	Coxiella
C_Sel18	Dikdik	Rainy	Fresh	Selous	Coxiella
C_Sel06	Gazelle	Rainy	Fresh	Selous	Coxiella
C_Sel07	Pig	Rainy	Fresh	Selous	Coxiella
C_Sel08	Cane Rat	Rainy	Fresh	Selous	Coxiella
C_Sel09	Buffalo	Rainy	Fresh	Selous	Coxiella
C_Sel19	Gazelle	Rainy	Fresh	Selous	Coxiella
C_Ser01	Eland	Rainy	Fresh	Serengeti	Coxiella
A_Sel03	Impala	Dry	Processed	Selous	Anthrax
A_Ser05	Pig	Dry	Processed	Serengeti	Anthrax
A_Ser01	Zebra	Rainy	Processed	Serengeti	Anthrax
A_Ser02	Zebra	Rainy	Processed	Serengeti	Anthrax
B_Sel01	Wildebeest	Rainy	Processed	Selous	Brucella
B_Ser05	Topi	Rainy	Processed	Serengeti	Brucella
B_Ser01	Impala	Rainy	Processed	Serengeti	Brucella
C_R01	Dikdik	Dry	Processed	Ruaha	Coxiella
C_R04	Dikdik	Rainy	Processed	Ruaha	Coxiella
