# Supplementary material for: Identification of Bacillus anthracis, Brucella spp., and Coxiella burnetii DNA signatures from bushmeat
Source: Sci Rep. 2021 Jul 21;11:14876. doi: 10.1038/s41598-021-94112-9 (PMC8295346; doi:10.1038/s41598-021-94112-9)
Supplement: Supplementary file 4 — Supplementary Information 4. [file 41598_2021_94112_MOESM4_ESM.pdf]

IDENTIFICATION OF *BACILLUS ANTHRACIS*, *BRUCELLA* SPP., AND *COXIELLA BURNETII* DNA SIGNATURES FROM BUSHMEAT

Robab Katani[1,2†], Megan A. Schilling[2,3†], Beatus Lyimo[4†], Ernest Eblate[4,5], Andimile Martin[4], Triza Tonui[6], Isabella M. Cattadori[2,4,7], Stephen C. Francesconi[8], Anna B. Estes[2,4**], Dennis Rentsch[9], Sreenidhi Srinivasan[2], Samson Lyimo[4], Lidia Munuo[4], Christian K. Tiambo[6], Francesca Stomeo[6***], Paul Gwakisa[10], Fausta Mosha[11], Peter J. Hudson[1,2,4,7], Joram J. Buza[4] and Vivek Kapur[1,2,3,4*]

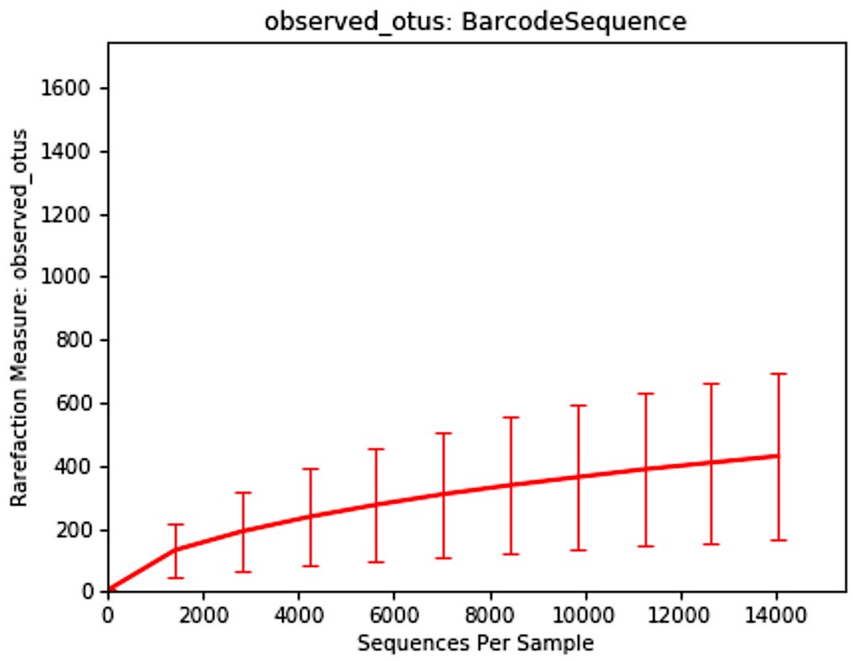

Supplemental Figure 1. Rarefaction Analysis. The curve illustrates the average OTUs observed (y-axis) from the sequences per sample (x-axis). The curve with standard deviation suggests that deeper or additional sequencing does not add significantly to the percentage of observed OTUs. It also indicates that differences in the number of sequences are not a significant contributor to the observed diversity in microbiota or the observed OTU in the samples.

IDENTIFICATION OF *BACILLUS ANTHRACIS*, *BRUCELLA* SPP., AND *COXIELLA BURNETII* DNA SIGNATURES FROM BUSHMEAT

Robab Katani[1,2†], Megan A. Schilling[2,3†], Beatus Lyimo[4†], Ernest Eblate[4,5], Andimile Martin[4], Triza Tonui[6], Isabella M. Cattadori[2,4,7], Stephen C. Francesconi[8], Anna B. Estes[2,4**], Dennis Rentsch[9], Sreenidhi Srinivasan[2], Samson Lyimo[4], Lidia Munuo[4], Christian K. Tiambo[6], Francesca Stomeo[6***], Paul Gwakisa[10], Fausta Mosha[11], Peter J. Hudson[1,2,4,7], Joram J. Buza[4] and Vivek Kapur[1,2,3,4*]

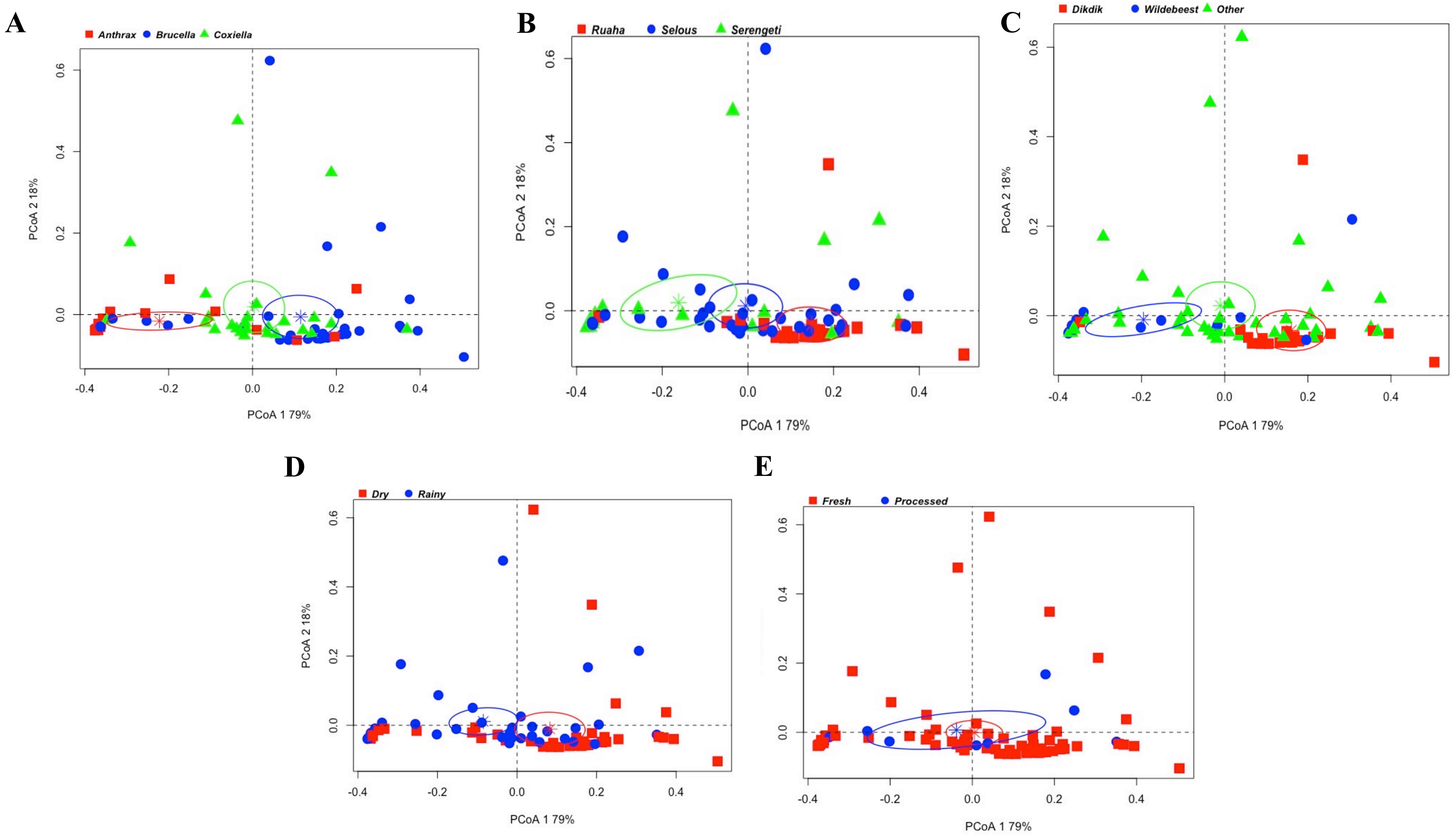

Supplemental Figure 2. The Principal Coordinates Analysis of the microbiota at the phylum level. The beta diversity of the samples was represented in Principal Coordinate Analysis. **(A)** The red squares represent *B. anthracis*-positive, the blue circles represent *Brucella*-positive, and the green triangles represent *Coxiella*-positive samples. **(B)** The same symbols represent the Selous, Ruaha, and Serengeti ecosystems, respectively. **(C)** The same symbols represent dikdik, wildebeest, and *Other* species, respectively. **(D)** The red squares represent dry season and the blue circles represent rainy season, and **(E)** The red squares represent fresh samples and the blue circles represents processed samples. The variability explained by the first two components are represented by the percentage on each axis. The ellipses represent the 95% confidence interval of the clustering in each respective color and the centers of the ellipses (or average value of the groups) are reported with the asterisk.

IDENTIFICATION OF *BACILLUS ANTHRACIS*, *BRUCELLA* SPP., AND *COXIELLA BURNETII* DNA SIGNATURES FROM BUSHMEAT

Robab Katani[1,2†], Megan A. Schilling[2,3†], Beatus Lyimo[4†], Ernest Eblate[4,5], Andimile Martin[4], Triza Tonui[6], Isabella M. Cattadori[2,4,7], Stephen C. Francesconi[8], Anna B. Estes[2,4**], Dennis Rentsch[9], Sreenidhi Srinivasan[2], Samson Lyimo[4], Lidia Munuo[4], Christian K. Tiambo[6], Francesca Stomeo[6***], Paul Gwakisa[10], Fausta Mosha[11], Peter J. Hudson[1,2,4,7], Joram J. Buza[4] and Vivek Kapur[1,2,3,4*]

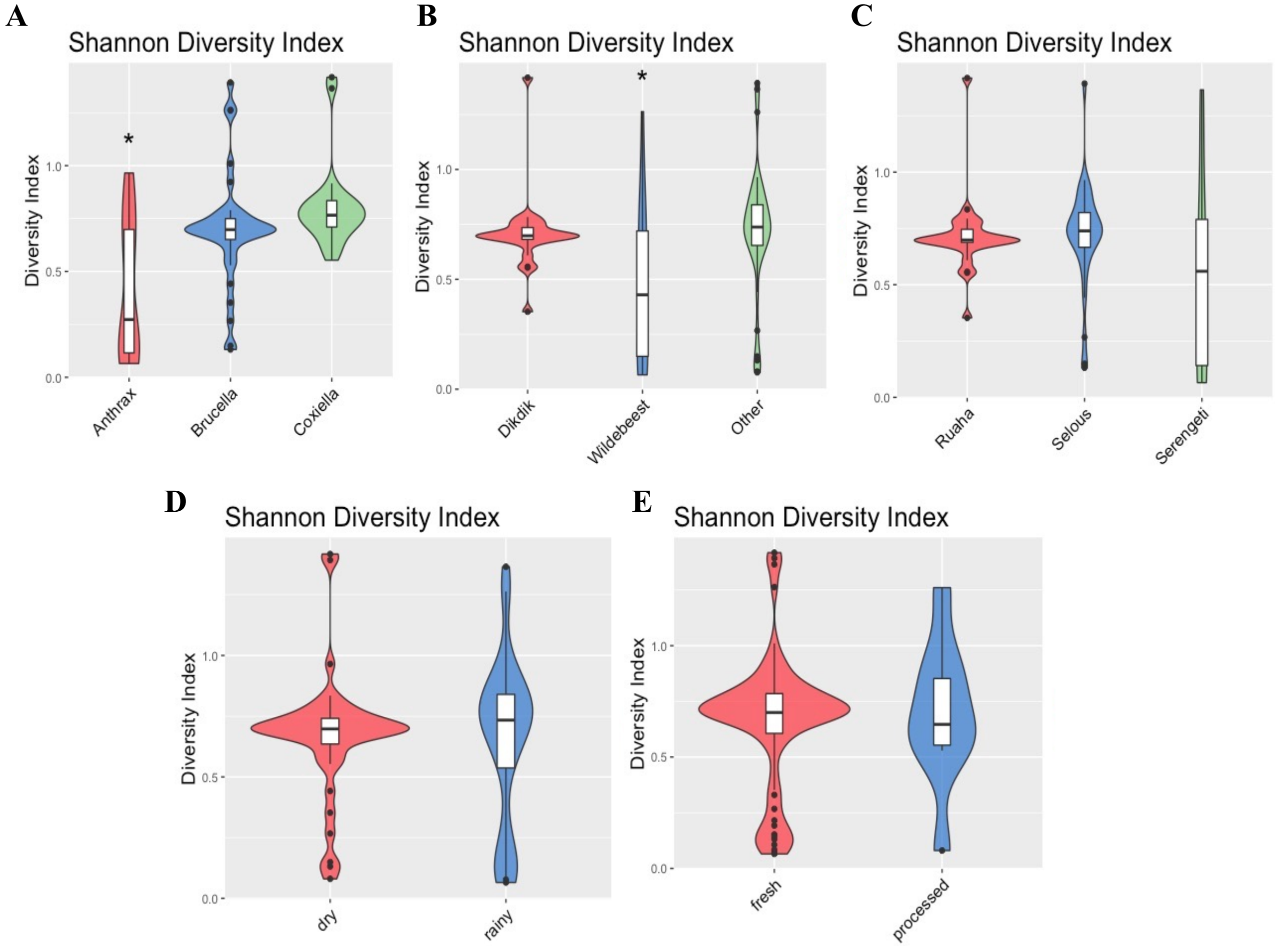

Supplemental Figure 3. Phylum-level alpha diversity. The Shannon Diversity Index (species richness) is visualized in the violin plots. The colored (violin) portion shows the distribution of the species richness. The boxplot shows the median (middle line), first and third quartiles (box), and range (whiskers). The dots show outliers. **A)** The species richness is significantly lower in the *B. anthracis*-positive samples (Red) (p-value < 0.001) as compared to the *Brucella*-positive samples (Blue) and *Coxiella*-positive samples (Green). **B)** The species richness is significantly lower in the wildebeest samples (Blue) (p-value < 0.001) as compared to the dikdik and *Other* species. No significant distribution in species richness is observed in samples with respect to region, season, and sample conditions **(C, D, E)**.

IDENTIFICATION OF *BACILLUS ANTHRACIS*, *BRUCELLA* SPP., AND *COXIELLA BURNETII* DNA SIGNATURES FROM BUSHMEAT

Robab Katani[1,2†], Megan A. Schilling[2,3†], Beatus Lyimo[4†], Ernest Eblate[4,5], Andimile Martin[4], Triza Tonui[6], Isabella M. Cattadori[2,4,7], Stephen C. Francesconi[8], Anna B. Estes[2,4**], Dennis Rentsch[9], Sreenidhi Srinivasan[2], Samson Lyimo[4], Lidia Munuo[4], Christian K. Tiambo[6], Francesca Stomeo[6***], Paul Gwakisa[10], Fausta Mosha[11], Peter J. Hudson[1,2,4,7], Joram J. Buza[4] and Vivek Kapur[1,2,3,4*]

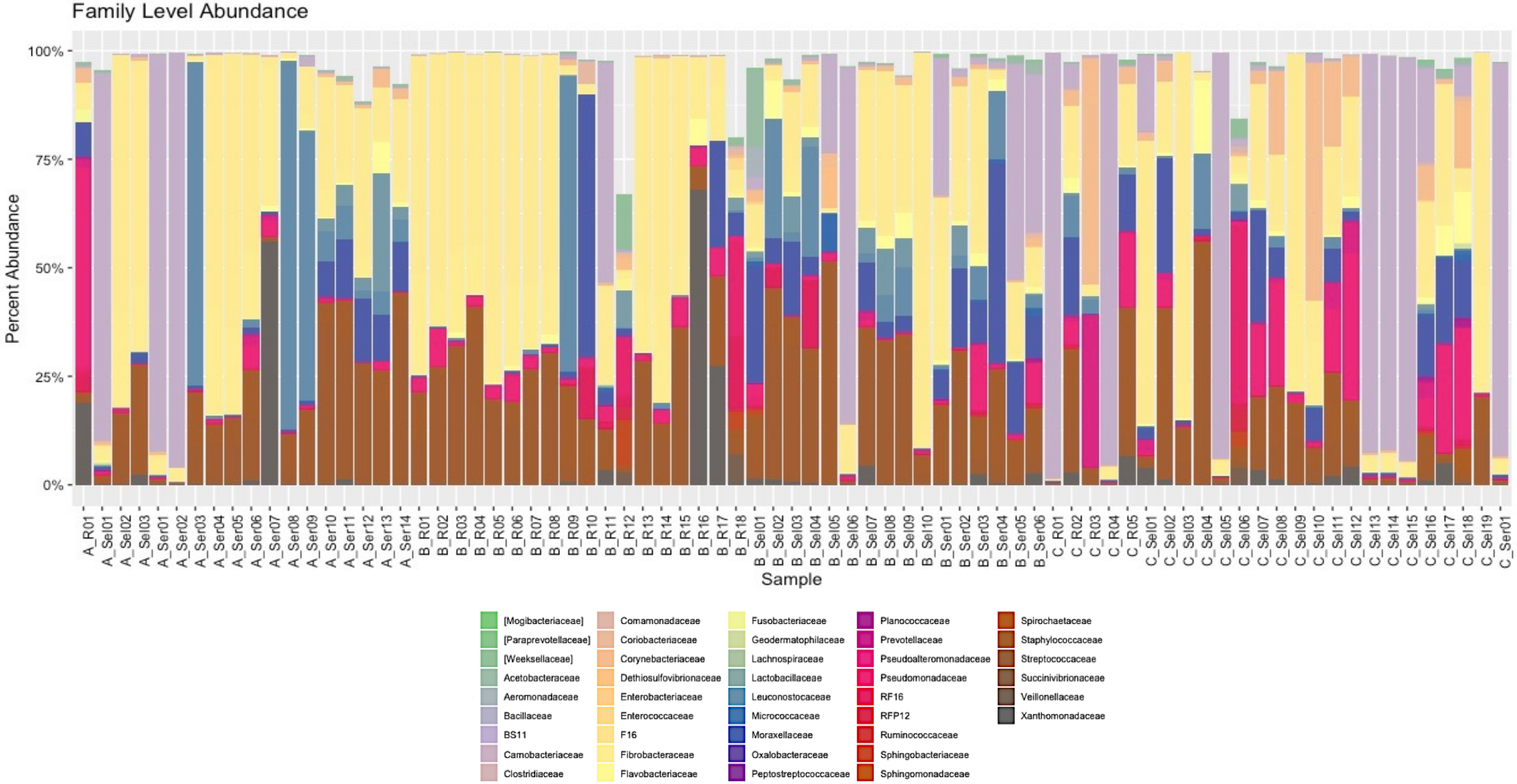

Supplemental Figure 4. The stacked bar graphs represent the relative abundance of taxa at the family level in each sample. *B. anthracis*-positive samples, *Brucella*-positive samples, and *Coxiella*-positive samples. The samples are identified by first letter of the pathogen followed by an underscore, name of the ecosystem they were collected from Ruaha (R), Selous (Sel), Serengeti (Ser), followed by sample number. The colors are representative of the respective family in the legend.

IDENTIFICATION OF *BACILLUS ANTHRACIS*, *BRUCELLA* SPP., AND *COXIELLA BURNETII* DNA SIGNATURES FROM BUSHMEAT

Robab Katani[1,2†], Megan A. Schilling[2,3†], Beatus Lyimo[4†], Ernest Eblate[4,5], Andimile Martin[4], Triza Tonui[6], Isabella M. Cattadori[2,4,7], Stephen C. Francesconi[8], Anna B. Estes[2,4**], Dennis Rentsch[9], Sreenidhi Srinivasan[2], Samson Lyimo[4], Lidia Munuo[4], Christian K. Tiambo[6], Francesca Stomeo[6***], Paul Gwakisa[10], Fausta Mosha[11], Peter J. Hudson[1,2,4,7], Joram J. Buza[4] and Vivek Kapur[1,2,3,4*]

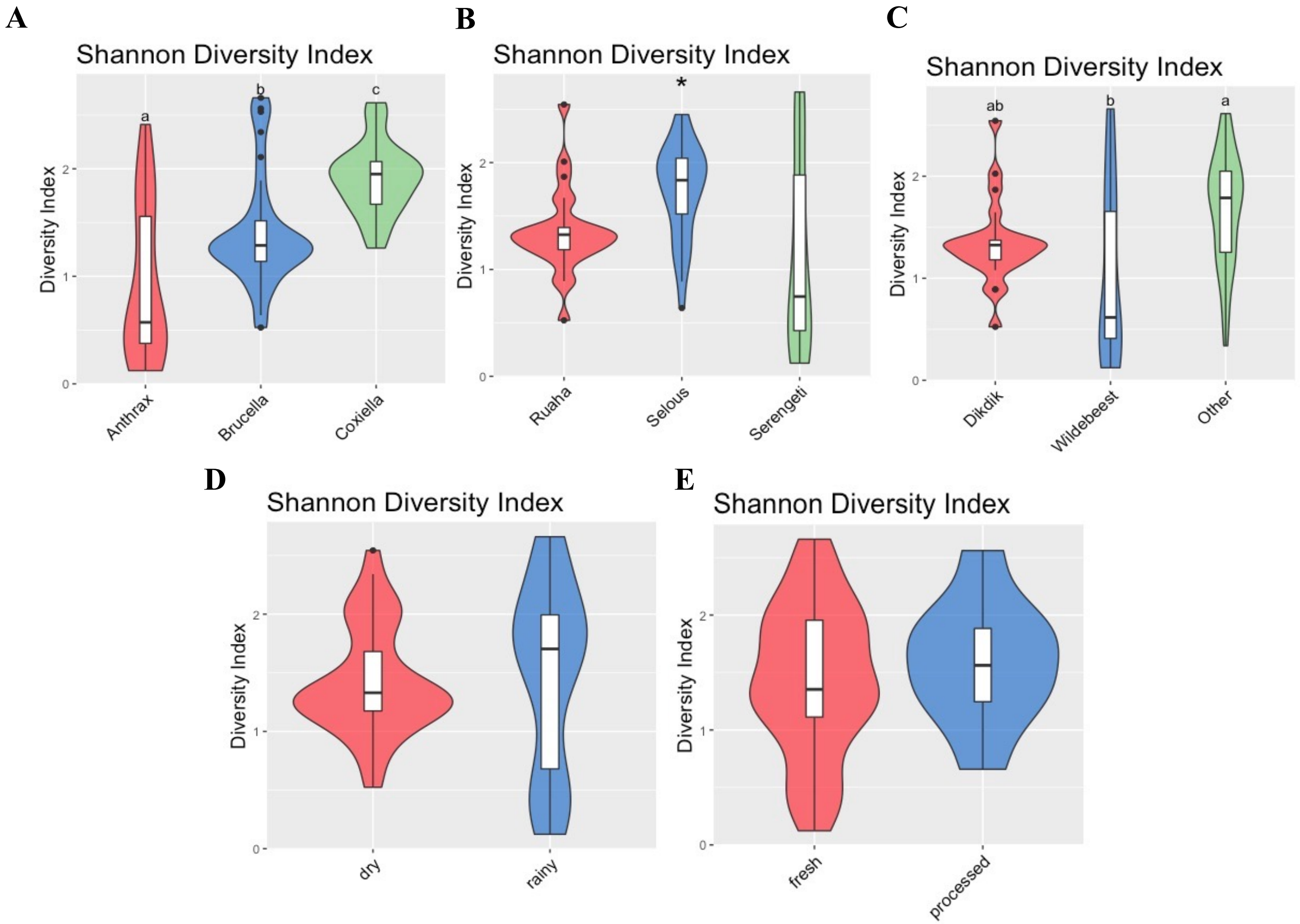

Supplemental Figure 5. Family-level alpha diversity. The Shannon Diversity Index (species richness) is visualized in the violin plots. The colored (violin) portion shows the distribution of the species richness. The boxplot shows the median (middle line), first and third quartiles (box), and range (whiskers). The dots show outliers. **A)** All *B. anthracis*- (Red), *Brucella*- (Blue) and *Coxiella*- (Green) positive samples are significantly different from each other (p-value < 0.001). **B)** The distribution of the species richness in the wildebeest (Blue) and *Other* (Green) are significantly different from each other and the dikdik (Red) samples are not significant from either. **(C-E)** No significant differences were observed in different regions, season, or samples condition at the family level.

IDENTIFICATION OF *BACILLUS ANTHRACIS*, *BRUCELLA* SPP., AND *COXIELLA BURNETII* DNA SIGNATURES FROM BUSHMEAT

Robab Katani[1,2†], Megan A. Schilling[2,3†], Beatus Lyimo[4†], Ernest Eblate[4,5], Andimile Martin[4], Triza Tonui[6], Isabella M. Cattadori[2,4,7], Stephen C. Francesconi[8], Anna B. Estes[2,4**], Dennis Rentsch[9], Sreenidhi Srinivasan[2], Samson Lyimo[4], Lidia Munuo[4], Christian K. Tiambo[6], Francesca Stomeo[6***], Paul Gwakisa[10], Fausta Mosha[11], Peter J. Hudson[1,2,4,7], Joram J. Buza[4] and Vivek Kapur[1,2,3,4*]

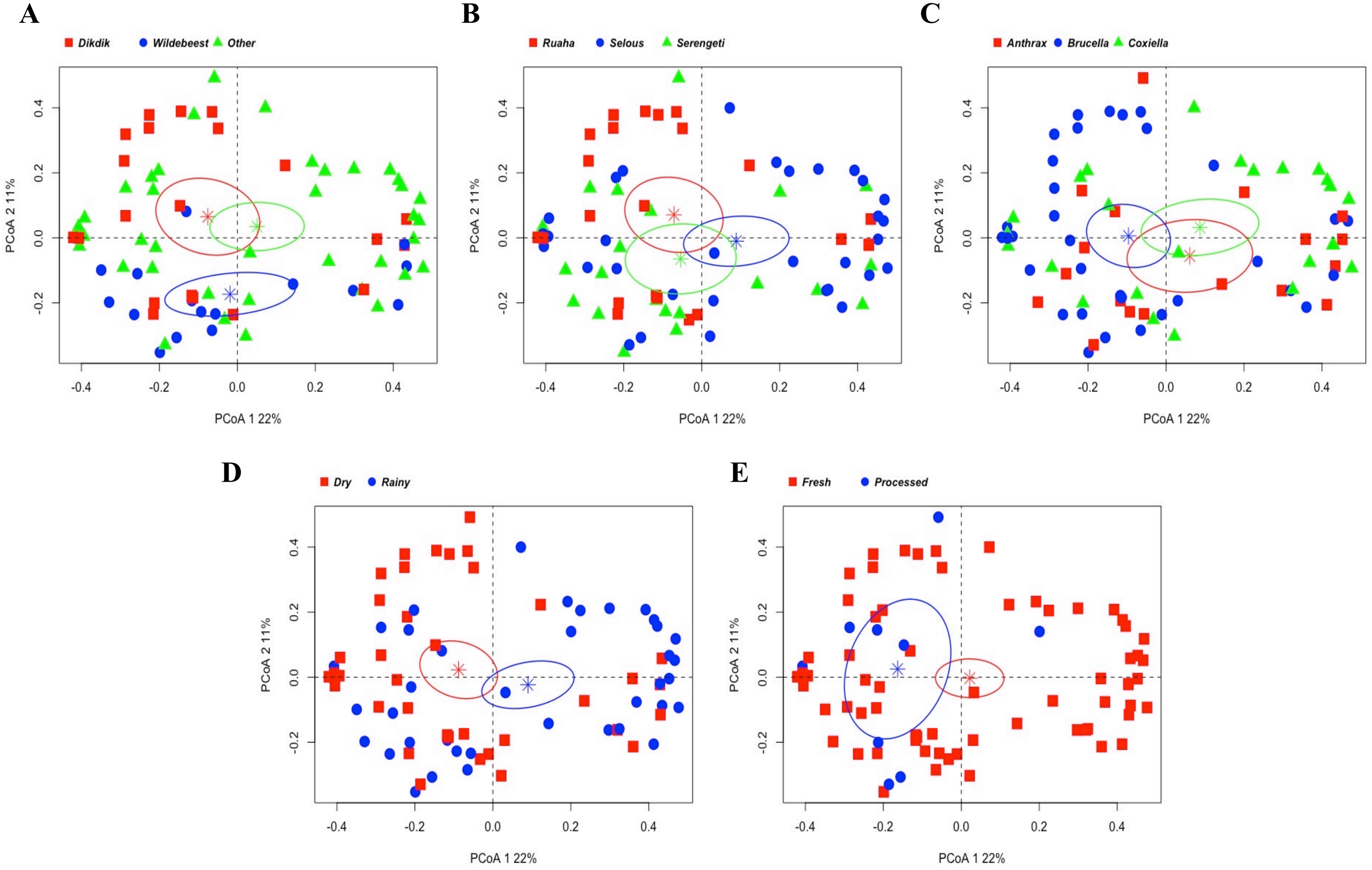

Supplemental Figure 6. The Principal Coordinates Analysis of the microbiota at the family level. The beta diversity of the samples was represented in Principal Coordinate Analysis. The legend at the top of each section shows the color and shape of each variable. **(A-B)** There is no distinct clustering associated with a specific pathogen or ecosystem**, (C)** There is a distinct clustering of the wildebeest samples compared to dikdik and *Other* samples, **(D-E)** Although there is a slight tendency of clustering with respect to season and sample condition. The variability explained by the first two components are represented by the percentage on each axis. The ellipses, represent the 95% confidence interval of the clustering, is represented as the respective colors in each sample series and the centers of the ellipses (or average value of the groups) are reported with the asterisk.

IDENTIFICATION OF *BACILLUS ANTHRACIS*, *BRUCELLA* SPP., AND *COXIELLA BURNETII* DNA SIGNATURES FROM BUSHMEAT

Robab Katani[1,2†], Megan A. Schilling[2,3†], Beatus Lyimo[4†], Ernest Eblate[4,5], Andimile Martin[4], Triza Tonui[6], Isabella M. Cattadori[2,4,7], Stephen C. Francesconi[8], Anna B. Estes[2,4**], Dennis Rentsch[9], Sreenidhi Srinivasan[2], Samson Lyimo[4], Lidia Munuo[4], Christian K. Tiambo[6], Francesca Stomeo[6***], Paul Gwakisa[10], Fausta Mosha[11], Peter J. Hudson[1,2,4,7], Joram J. Buza[4] and Vivek Kapur[1,2,3,4*]

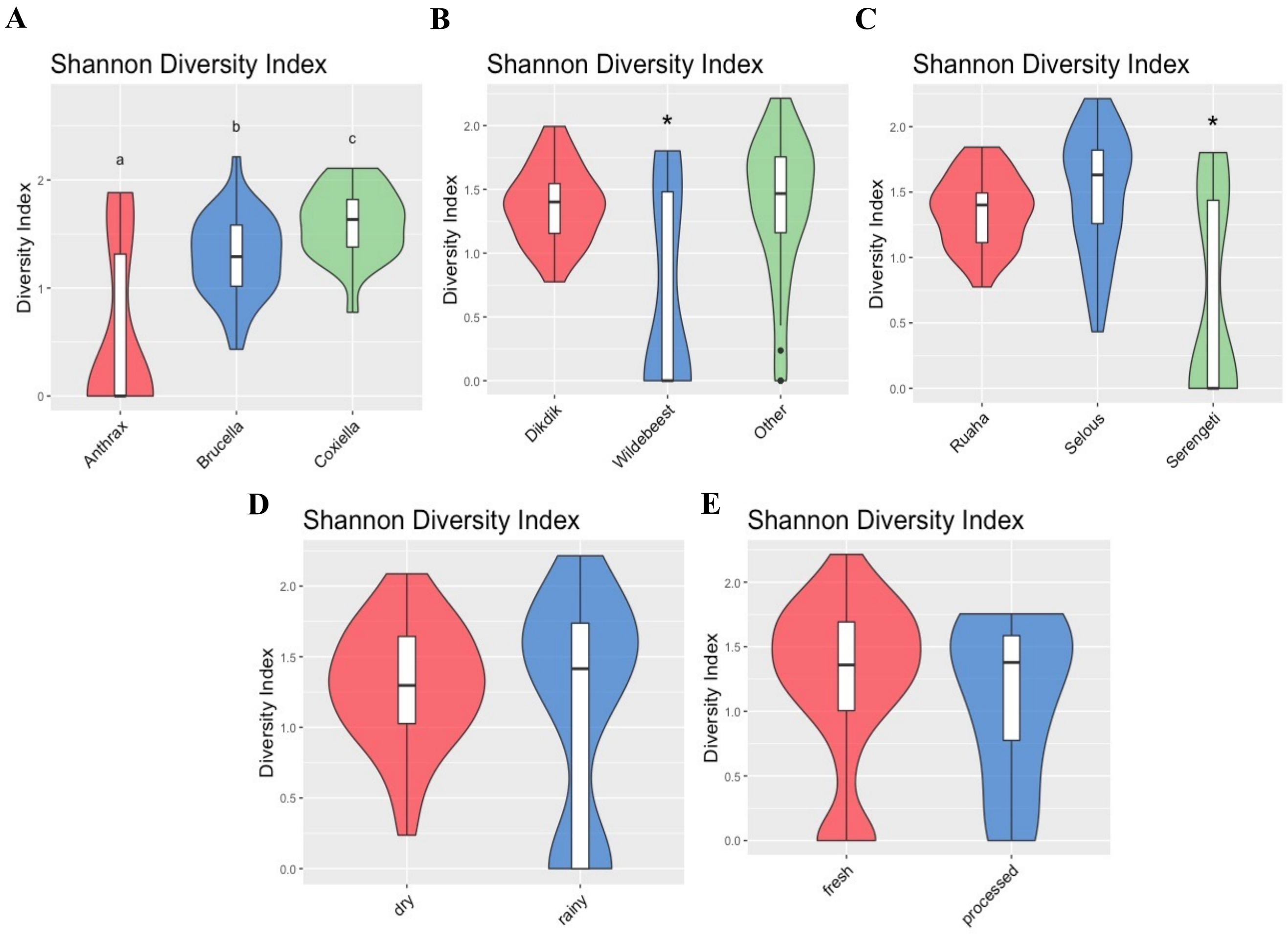

Supplemental Figure 7. Genus-level alpha diversity. The Shannon Diversity Index (species richness) is visualized in the violin plots. The boxplot shows the median (middle line), first and third quartiles (box), and range (whiskers). The dots show outliers. The colored (violin) portion shows the distribution of the species richness. **A)** The *Brucella*-positive samples have significantly lower diversity than the *Coxiella*-positive samples, however, the *B. anthracis*-positive samples are not significantly different than the others (p-value < 0.001). **D)** There is also a significantly lower species diversity in the dry season samples versus the rainy season. **B, C, E)** No significant differences were observed in different species, regions, or samples condition.

IDENTIFICATION OF *BACILLUS ANTHRACIS*, *BRUCELLA* SPP., AND *COXIELLA BURNETII* DNA SIGNATURES FROM BUSHMEAT

Robab Katani[1,2†], Megan A. Schilling[2,3†], Beatus Lyimo[4†], Ernest Eblate[4,5], Andimile Martin[4], Triza Tonui[6], Isabella M. Cattadori[2,4,7], Stephen C. Francesconi[8], Anna B. Estes[2,4**], Dennis Rentsch[9], Sreenidhi Srinivasan[2], Samson Lyimo[4], Lidia Munuo[4], Christian K. Tiambo[6], Francesca Stomeo[6***], Paul Gwakisa[10], Fausta Mosha[11], Peter J. Hudson[1,2,4,7], Joram J. Buza[4] and Vivek Kapur[1,2,3,4*]

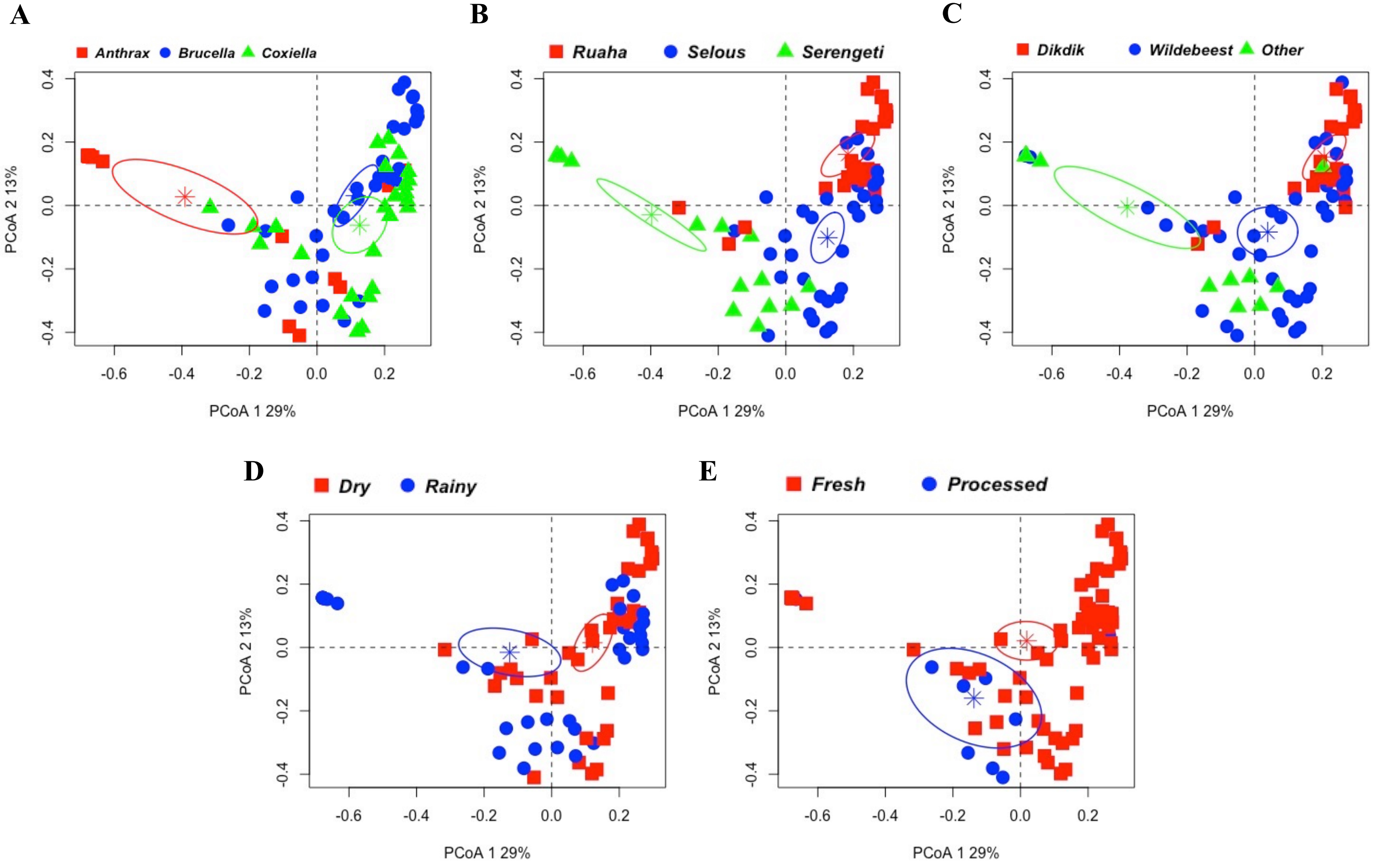

Supplemental Figure 8. The Principal Coordinates Analysis of the microbiota at the genus level. The beta diversity of the samples was represented in Principal Coordinate Analysis. **A)** The red squares represent Anthrax, the blue circles represent *Brucella*, and the green triangles represent *Coxiella*-positive samples. **(B)** The same symbols represent the Selous, Ruaha, and Serengeti ecosystems, respectively. **(C)** The same symbols represent dikdik, wildebeest, and Other species, **(D)** The red squares represents dry season and the blue circles represent rainy season, and **(E)** The red squares represents fresh samples and the blue circles represents processed samples. The variability explained by the first two components are represented by the percentage on each axis. The ellipses, represent the 95% confidence interval of the clustering in each respective color and the centers of the ellipses (or average value of the groups) are reported with the asterisk.
